# Single-Cell RNA Sequencing Revealed CD14^+^ Monocytes Increased in Patients With Takayasu’s Arteritis Requiring Surgical Management

**DOI:** 10.3389/fcell.2021.761300

**Published:** 2021-10-04

**Authors:** Gao Qing, Wu Zhiyuan, Yu Jinge, Miao Yuqing, Chen Zuoguan, Diao Yongpeng, Yin Jinfeng, Jia Junnan, Guo Yijia, Li Weimin, Li Yongjun

**Affiliations:** ^1^Graduate School of Peking Union Medical College, Chinese Academy of Medical Science, Beijing, China; ^2^Department of Vascular Surgery, National Centre of Gerontology, Beijing Hospital, Beijing, China; ^3^National Tuberculosis Clinical Lab of China, Beijing Chest Hospital, Beijing Tuberculosis and Thoracic Tumor Research Institute, Capital Medical University, Beijing, China; ^4^Beijing Key Laboratory in Drug Resistance Tuberculosis Research, Beijing Chest Hospital, Capital Medical University, Beijing, China; ^5^Institute of Statistics and Big Data, Renmin University of China, Beijing, China

**Keywords:** takayasu arteritis, single-cell RNA sequencing, monocytes, CD163, clinical marker

## Abstract

**Objectives:** Takayasu Arteritis (TA) is a highly specific vascular inflammation and poses threat to patients’ health. Although some patients have accepted medical treatment, their culprit lesions require surgical management (TARSM). This study aimed at dissecting the transcriptomes of peripheral blood mononuclear cells (PBMCs) in these patients and to explore potential clinical markers for TA development and progression.

**Methods:** Peripheral blood were collected from four TA patients requiring surgical management and four age-sex matched healthy donors. Single cell RNA sequencing (scRNA-seq) was adopted to explore the transcriptomic diversity and function of their PBMCs. ELISA, qPCR, and FACS were conducted to validate the results of the analysis.

**Results:** A total of 29918 qualified cells were included for downstream analysis. Nine major cell types were confirmed, including CD14^+^ monocytes, CD8^+^ T cells, NK cells, CD4^+^ T cells, B cells, CD16^+^ monocytes, megakaryocytes, dendritic cells and plasmacytoid dendritic cells. CD14^+^ monocytes (50.0 vs. 39.3%, *p* < 0.05) increased in TA patients, as validated by FACS results. TXNIP, AREG, THBS1, and CD163 increased in TA patients. ILs like IL-6, IL-6STP1, IL-6ST, IL-15, and IL-15RA increased in TA group.

**Conclusion:** Transcriptome heterogeneities of PBMCs in TA patients requiring surgical management were revealed in the present study. In the patients with TA, CD14^+^ monocytes and gene expressions involved in oxidative stress were increased, indicating a new treatment and research direction in this field.

## Introduction

Takayasu arteritis (TA), which prevails in East Asia, is a highly specific vasculitis that exclusively involves the large arteries and the main branches. In its early stage, patients with TA are barely manifest specific symptoms or signs, making the diagnosis very challenging. However, during the disease progression or in its late stage, the culprit lesion can lead to severe organ ischemia, such as cerebral infarction and myocardial infarction. This may be due to that these patients with TA in early stage are not timely diagnosed and treated.

Currently, medical treatment for TA mainly includes glucocorticoids (Comarmond et al., 2017), methotrexate (Hoffman et al., 1994), and mycophenolate mofetil (Li et al., 2016; Dai et al., 2017). In recent years, biological agents such as tocilizumab (Zhou et al., 2017) and infliximab (Torp et al., 2021) have also been used as candidate drugs for TA. However, the active inflammation of some patients cannot be effectively controlled following medical treatment; thus, the stenosis of the culprit vessels continues to progress, and these patients with TA ultimately requiring surgical management, including endovascular treatment and open surgical repair (Chen et al., 2018; Diao et al., 2020).

Recently, pathological studies investigated on the role of CD4^+^ T cells and interleukin (IL)-6 signaling pathway in the development and progression of TA (Sagar et al., 1992; Saadoun et al., 2015; Misra et al., 2016). IL-6 promotes the differentiation of CD4^+^ T cells into Th17 cells, which then secrete cytokines, such as IL-17, IL-21, and IL-22, and induce an autoimmune response (Ruzt et al., 2013; Sutherland et al., 2013; Camporeale and Poli, 2018). Current medical treatment are based on these mechanisms. However, as mentioned previously, it remains unclear why the culprit lesions still progress in patients with TA requiring surgical management (TARSM), even though these patients have already accepted medical drugs.

Nowadays, single-cell RNA sequencing (scRNA-seq), a high-throughput technology, has been utilized to dissect cellular heterogeneities in many immune diseases at the single-cell level (Papalexi and Satija, 2018; See et al., 2018). This new technology may provide a more precisely method to explore immune disease in different clinical stages. In this study, we adopted this state-of-the-art technique to dissect the transcriptomes of peripheral blood mononuclear cells (PBMCs) in patients with TARSM and explore potential clinical markers for the development and progression of TA.

## Materials and Methods

### Study Participants

Four female patients (27.75 ± 7.75 years old) who were admitted to Beijing Hospital Vascular Surgery Department from October 2019 to May 2020 were diagnosed with TA according to the American College of Rheumatology standard suggested by the American Rheumatism Association in 1990 (Arend et al., 1990). All patients including 3 active and 1 inactive had accepted medical treatment, but their clinical presentations still deteriorated and finally underwent surgical repair. Detailed clinical descriptions of the four patients and four age-sex-matched healthy donors are presented in Supplementary Table 1. The surgical classification of the patients with TA is also described in Supplementary Table 1. Peripheral blood samples were collected from the patients and healthy donors and used for scRNA-seq experiments. On the other hand, seven blood samples from outpatients with TA were collected for fluorescence-activated cell sorting (FACS), and four of the samples were used for quantitative PCR (qPCR).

Written consent was obtained from patients, healthy individuals, or their families. All the contents of this study met the relevant requirements of the ethics committee of Beijing Hospital. All experiments involving human samples were performed in accordance with the relevant regulations and current guidelines.

### Single-Cell Suspensions Preparation

**FIGURE 1:**
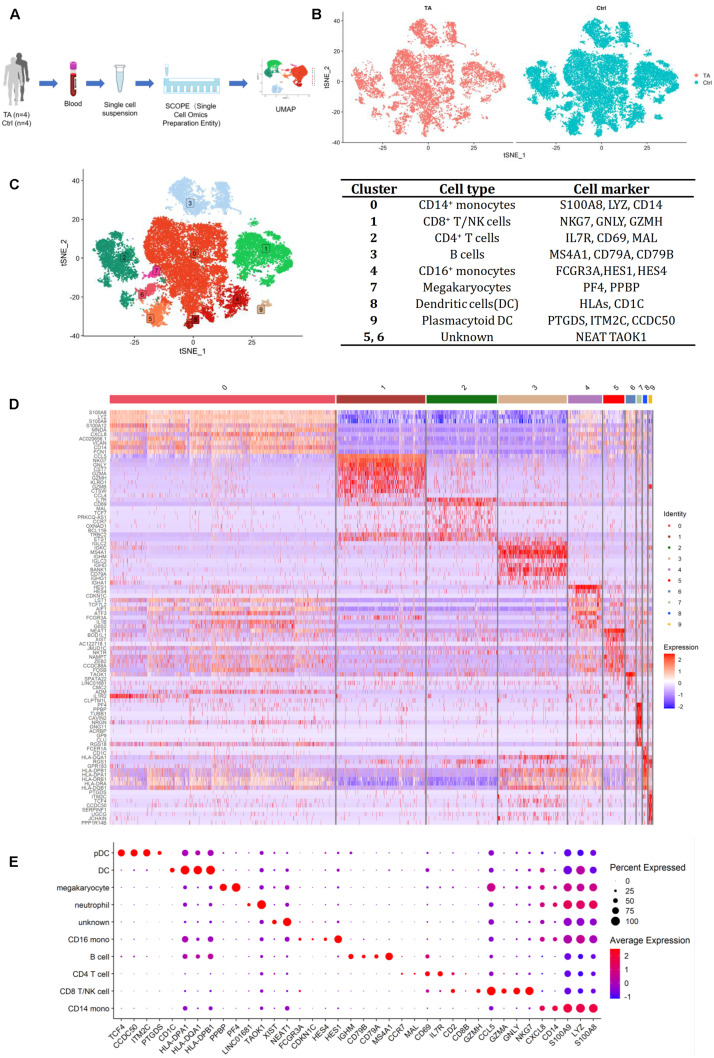
**(A)** The process of scRNA-seq. **(B)** Heterogeneity of TA and Ctrl group. **(C)** tSNE of 4 TA and 4 control samples and representative cell markers for each cluster, all cells were divided into 10 clusters. **(D)** Heatmap for 10 clusters. **(E)** Dotplot for 10 clusters. We chose 38 most representative markers for each cell type for dot plot construction. According to different expressed genes (DEGs) in heatmap, feature plots and dot plot, we verified CD14^+^ monocytes (cluster 0) by S100A8, LYZ, CD14; CD8^+^ T/NK cells (cluster 1) by NKG7, GNLY, GZMH; CD4^+^ T cells (cluster 2) by IL7R, CD69, MAL; B cells (cluster 3) by MS4A1, CD79A, CD79B; CD16^+^ monocytes (cluster 4) by HES1, HES4, FCGR3A; Megakaryocytes (cluster 7) by PF4, PPBP; Dendritic cells (cluster 8) by HLA-DPB1, HLA-DQA1, CD1C; Plasmacytoid dendritic cells (cluster 9) by PTGDS, ITM2C, CCDC50 and Undefined cells (cluster 5, 6) by NEAT1, XIST, TAOK1.

Density gradient centrifugation method was performed to obtain PBMCs. Phosphate buffered saline (PBS; Solarbio, P1022-500) was used to dilute the whole blood sample at a ratio of 1:1, and then the sample was added into a tube with approximately 2/3 volume of Ficoll (GE Healthcare, 17-1440-02). After centrifugation at 400× *g* for 35 min, three layers were obtained based on the size and density. The middle cell suspension layer was transferred into a new 15-ml centrifuge tube, added with PBS, and then centrifuged at 300× *g* for 7 min. The supernatant was discarded, the pellet containing PBMCs was washed twice and then resuspended in PBS to obtain a final concentration of 1 × 10^5^ cells/ml. Viability staining using 0.4% Trypan blue solution (Sigma, T8154) was performed, and viable cells were counted under a microscope. The experimental procedure is shown in Figure 1A.

### Single Cell RNA Sequencing

Single-cell suspensions were then loaded onto microfluidic devices, and scRNA-seq libraries were constructed according to the Singleron GEXSCOPETM protocol using the GEXSCOPETM Single-Cell RNA Library Kit (Singleron Biotechnologies). Individual libraries were diluted to 4 nM and pooled for sequencing. Pools were sequenced on an Illumina HiSeq X with 150 bp paired-end reads.

### Data Analysis

The Seurat package (v.4.0.1) (Butler et al., 2018; Stuart et al., 2019) was used for quality control (QC), processing, and analysis. Each Seurat object was generated with genes that were expressed in more than three cells. QC conditions were set as follows: (Comarmond et al., 2017) genes within 200 and 3,000, and (Hoffman et al., 1994) the percentage of mitochondrial genes less than 20% were included for downstream analysis. After QC, the remaining cells were used for further analysis. A total of 29,918 qualified cells were included in the computational analysis. Among them, the case (TA) group had 8,965 cells, and the healthy control (HC) group had 20,953 cells.

The CellCycleScoring function was used to calculate the cell cycle phase scores. SCTransform (Hafemeister and Satija, 2019) was adopted to reduce potential batch effects or technical variations. The principal component analysis was set at 1:20, and unsupervised cell clustering was performed.

The FindAllMarkers function with default settings was used to obtain the differentially expressed genes (DEGs) specific in each cluster, and the representative markers (genes with the high avg_logFC and adjusted *p*-value < 0.05) were then chosen for cluster labeling. DEGs between TA and HC in each cluster were identified using the FindMarkers function with the MAST method.

To determine the differences in cell composition between TA and HC, we used χ^2^ analyses to analyze the differences in the composition ratios of various cell types, and cells with higher composition ratios in TA were used. GraphPad Prism (v.8.0.2) was also used to plot the figures.

### Fluorescence-Activated Cell Sorting

FACS was used to test some significant markers and cell composition identified by scRNA-seq analysis. Another seven confirmed PBMCs from patients with TARSM were collected from Beijing Hospital outpatients. All samples were used for cell type and CD163 analyses. The antibodies included CD45 APC/Fire810 (HI30, 304076), CD3 FITC (SK7, 344803), CD4 PE/Cy7 (SK3, 344611), CD8 APC/Cy7 (SK1, 344713), CD19 PE/Dazzle 594 (SJ25C1, 363031), CD16 Brilliant Violet 650 (3G8, 302041), CD14 Brilliant Violet 785 (M5E2, 301839), CD56 Brilliant Violet 750 (5.1H11, 362555), and CD11c Alexa Fluor^®^ 647 (S-HCL-3, 371525). All antibodies were obtained from BioLegend. FACS was conducted using Cytek Aurora, and data were analyzed using SpectroFlo software (v.2.2.0.4).

### Enzyme-Linked Immunosorbent Assay

Serum samples were collected from TA and HC, and cytokine levels of thioredoxin-interacting protein (TXNIP), amphiregulin (AREG), Thrombospondin-1 (THBS1), and CD163 were measured using a 96T human ELISA kit (Dogesce, DG94224Q, DG96088Q, DG11739H, DG96191Q).

### Quantitative PCR

Total RNA was isolated from TA and HC groups using the RNAprep pure Cell/Bacteria Kit (TIANGEN, DP430), and reverse transcription was performed using the FastKing RT Kit (TIANGEN, KR116). Four mRNA genes (TXNIP, AREG, THBS1, and CD163) were amplified using qPCR, and the primer pairs were: TXNIP (Yang et al., 2019) (F: 5′-GCCACA CTTACCTTGCCAAT-3′; R: 5′-TTGGATCCAGGAACGCTA AC-3′), AREG (Hachim et al., 2020) (F: 5′-GAGCACCT GGAAGCAGTAAC-3’; R: 5′-GGATCACAGCAGACATAAA GGC-3′), THBS1 (Khosravi et al., 2019) (F: 5′-AGGACTG CGTTGGTGATGTA-3′; R: 5′-TCAGGCACTTCTTTGCACTC AT-3′), and CD163 (Sanhurjo et al., 2018) (F: 5′-CACCAGT TCTCTTGGAGGAACA-3′; R: 5′-TTTCACTTCCACTCTCC CGC-3′). The qPCR was conducted using SuperReal PreMix Plus (SYBR Green) (TIANGEN, FP205).

### Statistic Statement

We compared cell proportion of PBMCs between TARSM and healthy Ctrl. We also compared the levels of RNA and protein in these two groups. The statistical analysis of scRNA-seq were performed by R studio (v.1.2.1335) and results of cell proportion, ELISA, FACS and qPCR were managed by GraphPad Prism (v.8.0.2). All tests were two-sided and a *p*-value < 0.05 was considered to be significant.

## Results

### scRNA-Seq Analysis of Blood Samples

We analyzed PBMCs from four patients with TA and four healthy controls. After QC, a total of 29,918 qualified cells were included for downstream computational analysis, among which 8,965 cells were from TA group and 20,953 cells from HC group. A preliminary estimation of the cell composition in each sample revealed a similar distribution for each cluster (Figure 1B and Supplementary Figures 1A,B). Data from the eight samples were integrated for further analysis.

### Eight Major Cell Types

After unsupervised clustering, 10 clusters were initially obtained and identified by typical markers highly expressed in each cluster, including CD14^+^ monocytes (cluster 0), CD8^+^ T/natural killer (NK) cells (cluster 1), CD4^+^ T cells (cluster 2), B cells (cluster 3), CD16^+^ monocytes (cluster 4), megakaryocytes (cluster 7), dendritic cells (cluster 8), plasmacytoid dendritic cells (cluster 9), and undefined cells (clusters 5 and 6) (Figures 1C–E, Supplementary Figure 1C, and Supplementary Table 2).

**FIGURE 2:**
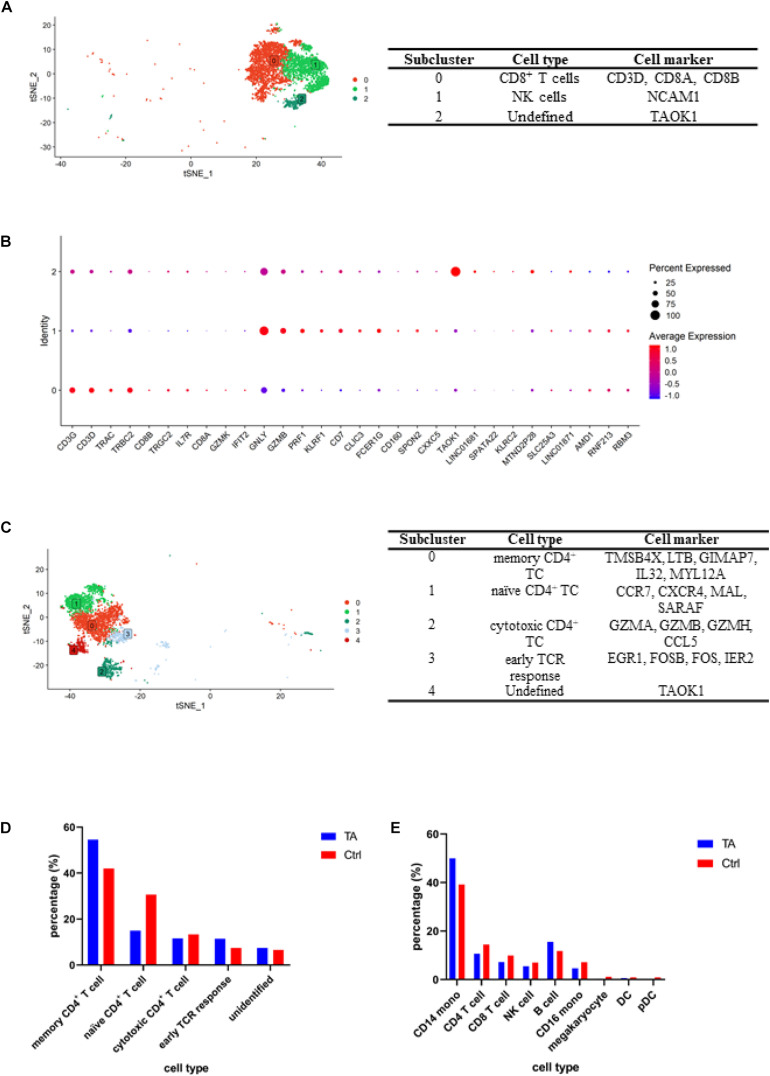
**(A)** tSNE for CD8^+^ T/NK cell clusters and representative cell markers for each cluster. **(B)** Dotplot for CD8^+^ T/NK cell clusters. To further separate CD8^+^ T cells and NK cells, we conducted an independent analysis for cluster 1, and according to the expression of specific genes, including CD8A, CD8B, NCAM1 (CD56), we obtained CD8^+^ T cells (subcluster 0) and NK cells (subcluster 1). **(C)** tSNE for CD4^+^ T cell clusters and representative cell markers for each cluster. We verified memory CD4^+^ T cell (subcluster 0) by TMSB4X, LTB, GIMAP7, IL32, MYL12A; naïve CD4^+^ T cell (subcluster 1) by CCR7, CXCR4, MAL, SARAF; cytotoxic CD4^+^ T cell (subcluster 2) by GZMA, GZMB, GZMH, CCL5; early TCR response (subcluster 3) by EGR1, FOSB, FOS, IER2. **(D)** Comparation of CD4^+^ T cells composition between TA and Ctrl group. It can be found the percentages of memory CD4^+^ T cell were higher in TA than Ctrl. **(E)** Comparation of each cell type percentage between TA and Ctrl group and CD14^+^ monocyte and B cell were higher in TA than Ctrl.

To distinguish CD8^+^ T cells and NK cells, we further analyzed cells from Cluster 1 (Figure 2A and Supplementary Table 3) and obtained CD8^+^ T cells (subcluster 0) and NK cells (subcluster 1) according to the gene expression of each subcluster (Figure 2B and Supplementary Figure 2A).

Analysis of CD4^+^ T cells was also conducted (Figure 2C and Supplementary Table 4). According to the gene expression of each subcluster, we obtained five cell populations, including memory CD4^+^ T cells (subcluster 0) using TMSB4X, LTB, GIMAP7, IL32, and MYL12A; naïve CD4^+^ T cells (subcluster 1) using CCR7, CXCR4, MAL, and SARAF; cytotoxic CD4^+^ T cell (subcluster 2) using GZMA, GZMB, GZMH, and CCL5; early TCR response (subcluster 3) using EGR1, FOSB, FOS, IER2; and unidentified cluster using TAOK1 and LINC01681 (Ding et al., 2020; Figure 2C and Supplementary Figure 2).

### Cellular Proportions of TARSM and HC

Next, we compared the cellular proportions between TA and HC groups. As shown in Figure 2D, compared with those in HC group, memory CD4^+^ T cells in TA group increased (54.57 vs. 42.04%, *p* < 0.05). Meanwhile, the proportion of naïve CD4^+^ T cells (14.93 vs. 30.62%, *p* < 0.05) and cytotoxic CD4^+^ T cell decreased (11.57 vs. 13.32%, *p* < 0.05), moreover, CD4^+^ T cells (10.6 vs. 14.4%, *p* < 0.05), CD8^+^ T cells (7.2 vs. 9.9%, *p* < 0.05), and NK cells (5.5 vs. 7.0%, *p* < 0.05) (Figure 2E).

The data also revealed that CD14^+^ monocytes (50.0 vs. 39.3%, *p* < 0.05) and B cells increased significantly (15.5 vs. 11.8%, *p* < 0.05), while CD16^+^ monocytes (4.6 vs. 7.1%, *p* < 0.05), megakaryocytes (0.3 vs. 1.1%, *p* < 0.05), dendritic cells (0.6 vs. 0.9%, *p* < 0.05), and plasmacytoid dendritic cell (0.2 vs. 0.9%, *p* < 0.05) populations decreased in patients with TARSM.

### Transcriptomics Altered in TARSM

**FIGURE 3:**
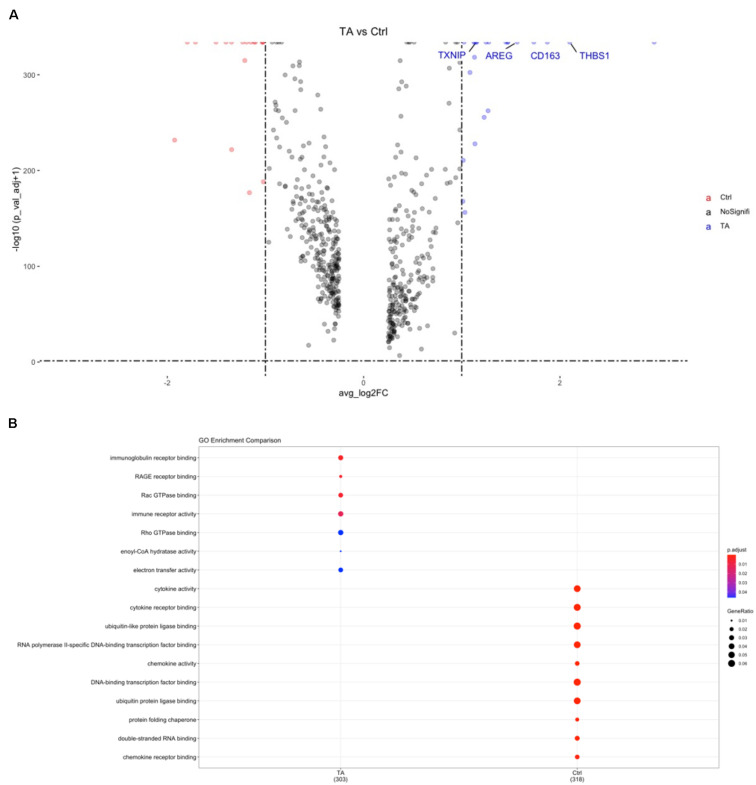
**(A)** Volcano plot for gene expression of cell markers in PBMCs between TA and Ctrl group. TXNIP, AREG, THBS1, CD163 were significantly higher in TA group. **(B)** GO analysis of CD14^+^ monocytes. RAGE pathway can be found in TA group.

We then explored DEGs between the two groups. In TA group, 251 genes were significantly highly expressed (avg_logFC > 0.25 and adjusted *P*-value < 10^–10^; Supplementary Table 5). We also performed the same analysis in each cell type to explore the DEGs between the TA and HC groups. Four differentially expressed genes (TXNIP, AREG, THBS1, and CD163) were selected for further analysis (Figure 3A). Results revealed that CD163, AREG, THBS1, and TXNIP have higher expression in CD14^+^ and CD16^+^ monocytes.

We also performed gene orthology (GO) analysis in each cluster, and pathway analysis based on the DEGs was conducted using GO in CD14^+^ monocytes. Among these, binding of the receptor for advanced glycation end products (RAGE) was observed in CD14^+^ monocytes (Figure 3B) and dendritic cells (DCs) (Supplementary Figure 3A), indicating that this receptor pathway may be widely activated in antigen-presenting cells (APCs) of patients with TARSM.

### Interleukin Family Genes in TARSM

**FIGURE 4:**
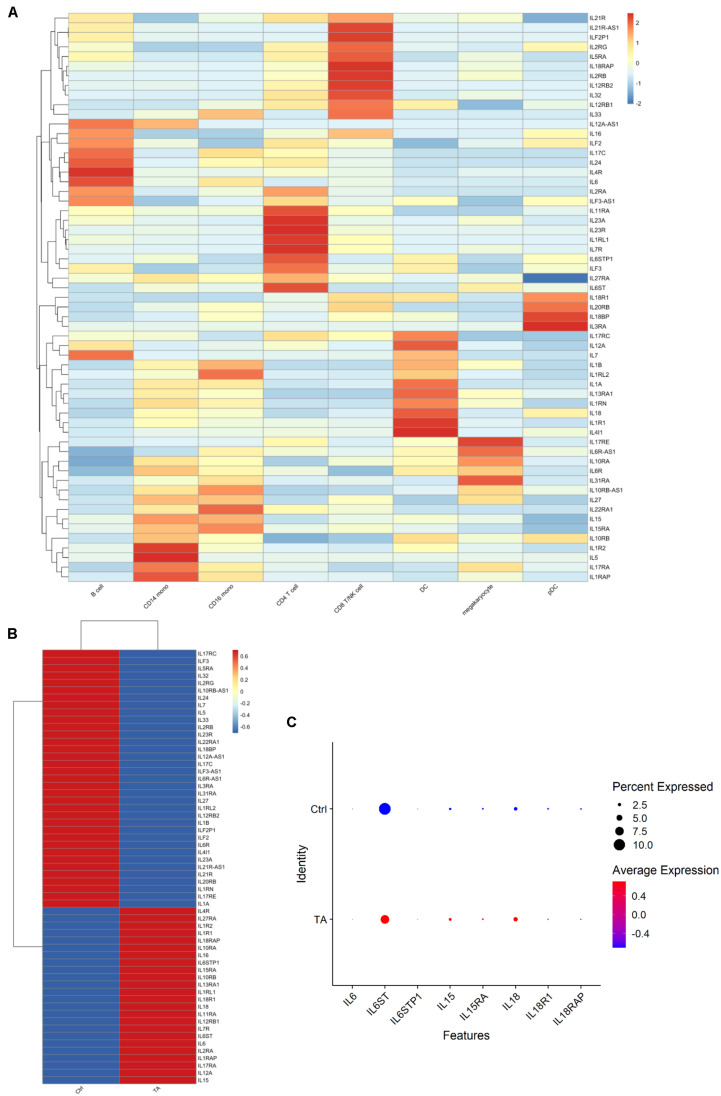
**(A)** The expression of ILs in each cell type. **(B)** The expression of ILs in TA and Ctrl group. **(C)** Dotplot of 8 IL genes. It can be found IL-6, IL-6STP1, IL-6ST, IL-15, IL-15RA, IL-18, IL-18RAP, and IL-18R1 were detected in TA group.

Since IL family genes play significant roles in TA diseases (Mirault et al., 2017), we next explored how ILs changed from HC group to TA group. We listed the IL- or IL-related genes included in our datasets and explored their expression in the cell types among PBMCs. As shown in Figure 4A, each cell type possessed highly expressed IL-related gene clusters. For example, IL-1R2, IL-5, IL-17RA, and IL-1RAP were highly expressed in CD14^+^ monocytes. Then, we also compared the expression of these genes between the two groups. We observed that some IL genes were highly expressed in TA group such as IL-6, IL-6STP1, IL-6ST, IL-15, IL-15RA, IL-18, IL-18RAP, and IL-18R1 (Figures 4B,C). We also observed that IL-6 was highly expressed in B cells and CD16^+^ monocytes. In addition, IL-6 levels significantly increased in CD16 monocytes of TA group (Supplementary Figure 3B). However, how the IL genes contribute to the progression of TA remains to be elucidated.

### FACS Revealed Similar Results

**FIGURE 5:**
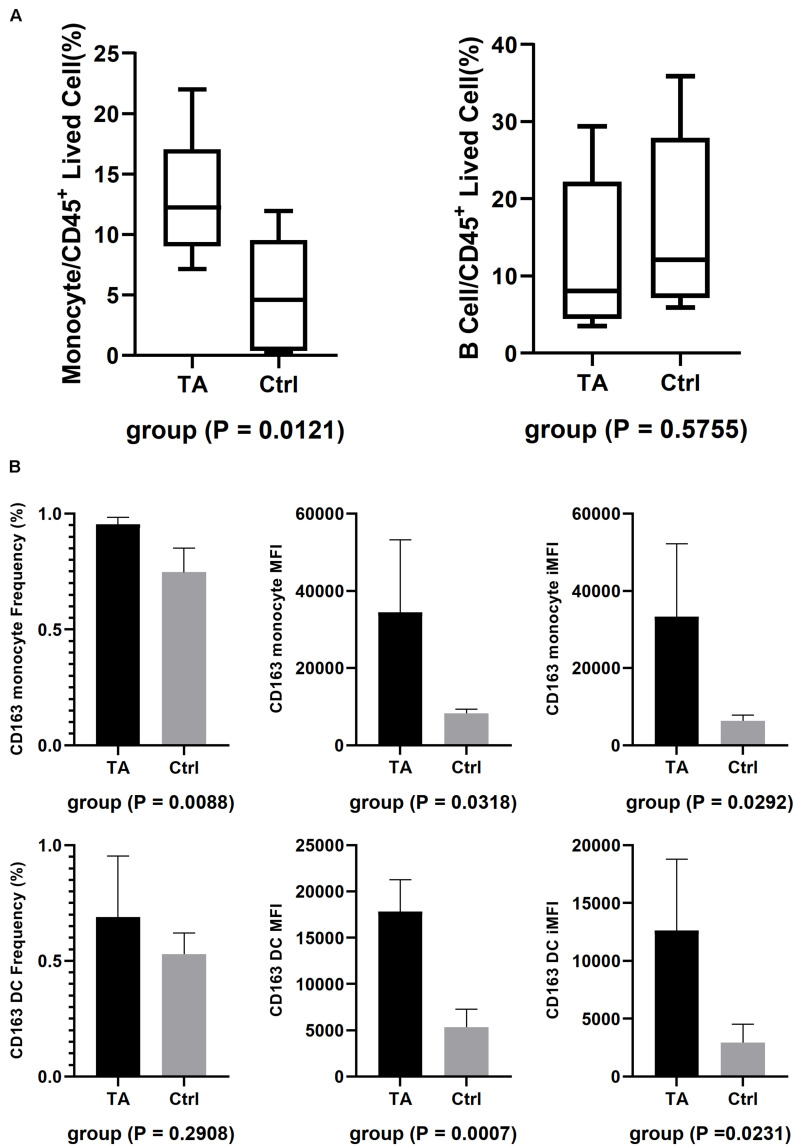
FACS results. **(A)** The composition of CD14^+^ monocyte and B cell in TA and Ctrl group. Only CD14^+^ monocyte had higher composition in TA than Ctrl. **(B)** iMFI showed both monocytes and DC had a higher expression on their membrane in TA group.

We then utilized FACS to validate our observations from the scRNA-seq results. First, different cell types were used in this study, and the results are shown in Figure 5 and Supplementary Figure 4. Nine antibodies were used for identification (Supplementary Figure 4A). We compared the proportion of monocytes and B cells between TA and HC groups and observed that the proportion of monocytes increased (13.34 ± 5.307% vs. 5.31 ± 4.836%, *p* = 0.0121), but the difference in that of B cells was not significant (12.40 ± 9.822% vs. 15.71 ± 11.60%, *p* = 0.5755) in TA group (Figure 5A). Then, we used the integrated median fluorescence intensity (iMFI) value (Darrah et al., 2007) to compare and assess the expression of CD163 in the membrane of APCs (Supplementary Figures 4B,C) and found that CD163 was highly expressed in both monocytes (33,272 ± 18,904 vs. 6,252 ± 1,505, *p* = 0.0292) and DCs (12,619 ± 6,188 vs. 2,943 ± 1,580, *p* = 0.0231) in TA group (Figure 5B).

### Quantitative PCR and ELISA

We also evaluated the expression of TXNIP, AREG, THBS1, and CD163. First, qPCR was performed, as shown in Supplementary Figure 5A. All four genes showed higher expression in TA group than in HC group, which was consistent with scRNA-seq. Then, we validated their expression by ELISA, as shown in Supplementary Figure 5B. Results showed that the levels of TXNIP (3.862 ± 0.4976 ng/ml vs. 3.478 ± 0.3369 ng/ml) and AREG (186.4 ± 26.33 pg/ml vs. 168.5 ± 20.34 pg/ml) were higher in TA group, which was similar to scRNA-seq results. However, THBS1 (64.32 ± 13.48 ng/ml vs. 69.38 ± 4.524 ng/ml) and CD163 (122.4 ± 10.21 ng/ml vs. 124.9 ± 11.28 ng/ml) levels were lower, indicating they were not increased in serum.

## Discussion

At present, research on the pathogenesis of TA has mainly focused on genes (Terao, 2016) and proteins (Mirault et al., 2017) of the peripheral blood of patients, especially in T cells (Regnier et al., 2020). However, it remains unclear how the other types of inflammatory cells in the peripheral blood contribute to the progression and development of TA. Kotaro found that IL-1 pathway expression was elevated in the peripheral blood of patients with refractory large vessel vasculitis by analyzing bulk-seq data, but the author failed to elucidate the relationship between the IL-1 pathway and different types of immune cells (Matsumoto et al., 2021). Compared with bulk-seq, scRNA-seq technology can analyze the transcriptome in each cell type, which provides more comprehensive information on the function of different inflammatory cells. To the best of our knowledge, this is the first time that scRNA-seq was used to detect the pathogenesis in the peripheral blood of patients with TA and identify potential cell markers.

In this study, it was revealed that the proportion of monocytes in patients with TARSM was higher in TA group than in HC group, which was similar in other immune-related diseases such as tuberculosis (Naranbhai et al., 2014), rheumatoid arthritis (Du et al., 2017), and vitiligo (Demirbas et al., 2020). This phenomenon observed in this study might be due to the following mechanism: **(a)** CD14^+^ monocytes trigger immune responses, and **(b)** CD163 can inhibit the activation and proliferation of lymphocytes and reduce the absolute value of lymphocytes (Baeten et al., 2004). CD163 is a transmembrane scavenger receptor (Law et al., 1993) and is known to be elevated in patients with systemic lupus erythematosus (Borgia et al., 2018), systemic juvenile idiopathic arthritis (Minoia et al., 2015), and Kawasaki disease (Garcia-Pavon et al., 2017). CD163 is also considered as a potential marker for macrophage activation syndrome, which promotes the transition from monocytes/macrophages to M1 proinflammatory macrophages (Porcheray et al., 2005; Avcin et al., 2006). In the present study, the elevated CD163 in APC membranes in patients with TA indicates that these cells are in an active state of inflammation, suggesting that APCs may play an important role in patients with TARSM and they are activated even earlier than CD4^+^ T cells.

Other cell markers highly expressed in the APCs of patients with TA were also analyzed, including TXNIP, THBS1, and AREG. Among these three markers, TXNIP and AREG showed consistent results in ELISA. TXNIP is a binding protein of thioredoxin (TXN) and can inhibit the antioxidant capacity of TXN and promote cell stress (Tinkov et al., 2018). TXNIP promotes the formation of reactive oxygen species (ROS)-NLRP3 inflammasomes by inhibiting the transfer of ROS by TXN, thereby increasing the concentration of IL-18 (Kim et al., 2019), which has also been shown to increase in patients with TARSM (Alibaz-Oner et al., 2015). NLRP3 inflammasomes are mainly secreted by activated macrophages (Kelley et al., 2019). At the same time, TXNIP is a negative regulator of thioredoxin, a key antioxidant protein to remove reactive oxygen and promote DNA repair (Lu and Holmgren, 2014). It means the high expression of TXNIP would results in a severe oxidative stress in the patient’s body (Yu et al., 2013). The increased expression of TXNIP in TARSM monocytes is closely related to their function.

AREG, as one of the main ligands of the EFGR pathway, mainly participates in the regulation of proliferation, apoptosis, and metastasis of various cells (Berasain and Avila, 2014). AREG expression is mainly elevated in pathological conditions, such as cirrhosis (Perugorria et al., 2008) and chronic obstructive pulmonary disease (Val et al., 2012). Recent studies on mouse models of glomerulonephritis also showed that AREG can enhance the function of Treg cells, inhibit the growth of CD4^+^ T cells, and promote the recruitment of myeloid cells, proliferation, and cytokine secretion of M1 cells (Melderis et al., 2020). The increase in AREG in patients with TARSM might serve as an activation signal for monocytes.

Recent studies have shown the important roles of IL family genes in the progression of TA. Among them, IL-6 has received the most attention (Sagar et al., 1992), and anti-IL-6R biologics have also been widely used in clinical practice (Zhou et al., 2017). In this study, IL-6 was not only highly expressed in APCs, but also in B cells and even autocrine in CD4^+^ T cells, indicating that B cells and CD4^+^ T cells may have other unknown functions in TA. Interestingly, IL-15 and its receptor gene IL-15RA, which are widely expressed in a variety of cells (Patidar et al., 2016) and play a bridge between innate immunity and adaptive immunity (Pagliari et al., 2013), were also detected in our study. IL-15 can also activate the maturation and functional expression of T cells, DCs, and NK cells (Mattei et al., 2001; Saikh et al., 2001; Ali et al., 2015). In this study, IL-15 and its receptor IL-15RA were both highly expressed in APCs, indicating that their antigen presentation function is activated. IL-18 is mainly synthesized by APCs; meanwhile, its receptor genes, IL-18Rα and IL-18Rβ, are expressed in T cells and DCs. The main function of this pathway is to activate the NF-κB pathway and promote the synthesis of IFN-γ in Th1 cells (Nakanishi et al., 2001; Dinarello et al., 2013; Turner et al., 2014). As previously mentioned, IL-18 has been proven to be elevated in patients with TA (Minoia et al., 2015). In this study, IL-18 was found to be highly expressed in the DCs of patients with TA, and its receptor gene was highly expressed in CD8^+^ T/NK cells, indicating that the IL-18 pathways may also occur in the PBMCs of patients with TARSM.

RAGE is a cell transmembrane receptor used to recognize and bind AGEs (Schmidt et al., 1992). AGEs refer to a kind of glycosylated molecules produced by oxidative stress or metabolism, which include not only cytokines but also some metabolites. After RAGE recognizes and binds to the corresponding ligand, it can activate the NF-κB pathway, thereby promoting the secretion of cytokines by inflammatory cells, such as IL-6 and TNF-α (Shen et al., 2020). By the way, the activation of RAGE and NFκB also send a message to reactive oxidant species (ROS), a key role in oxidative stress, by NADPH oxidase (Daffu et al., 2013). In this study, according to the GO analysis results of different cell types, it was found that APCs, such as CD14^+^ monocytes and DCs, have prominent expression of RAGE pathways, indicating that activation of this pathway may be an important reason for APC activation.

Both TXNIP and RAGE are closely related to oxidative stress. Oxidative stress has been proposed as a root cause in development of many cardiovascular diseases, including atherosclerotic (Marchio et al., 2019), aneurysm (Emeto et al., 2016) and even TA (Mahajan et al., 2010). In human vessel, endothelial cells have become the major character of antioxidative, and the secreted endothelial nitric oxide synthase (eNOS) can produce NO, a vasoprotective molecule that can resist oxidation and inhibit the immune response of blood vessel walls (Forstermann et al., 2017). However, as the body is in an immune response or infection state, NADPH oxidase produced by immune cells can directly act on endothelial cells, destroy the stability of eNOS and inhibit the activity of NO, which finally produce a large amount of superoxide anion (Ghosh et al., 2017). The high expression of TXNIP and RAGE in monocyte suggested that oxidative stress may also be a cause of vascular intimal damage, and the trigger role played by monocyte cannot be ignored.

As previously mentioned, this is the first study to explore transcriptomics using scRNA-seq techniques in patients with TARSM. However, some limitations of this study should not be ignored. First, it is hardly to find TARSM patients without medical treatment for the following reasons: **(a)** For these patients, they are still unable to control the inflammatory activity even after receiving medical treatment, causing the disease progressing, and the vascular lumen continues to narrow, which finally to be occluded, then surgical treatment is required. **(b)** For the treatment strategy, medical therapy is still the first choice for TA treatment, while surgical operation is mainly for patients who have complications such as insufficient blood supply due to vascular occlusion. Second, the number of patients in this study was also limited, both in the scRNA-seq studies and in the validation studies, such as ELISA, qPCR, and FACS. In addition, the scRNA-seq technique adopted in this study only reflected a snapshot scene for this complex disease, and how the culprit lesion evolves requires further demonstration by longitudinal studies. Another issue that remains to be elucidated is the role of ILs in TA; that is, the correlation between the observation of ILs in this study and clinical studies needs to be explored and established. A validation cohort from another center would be an important next step in future research.

## Conclusion

In conclusion, we used single-cell RNA technology to detect peripheral blood cells in patients with TARSM. Our study showed that the proportion of CD14^+^ monocytes, function, and functional receptors increased in patients with TARSM after medical treatment, which suppressed CD4^+^ T cell function. Moreover, monocytes have become a major factor in inflammation, and the inhibition of monocyte population and function can be used as a new direction for medical treatment. We also found that TXNIP and AREG can be used as diagnostic markers for TA development and progression, and highly expressed CD163 may be an important characteristic of APCs in patients with TARSM.

## Data Availability Statement

The data presented in the study are deposited in the Genome Sequence Archive for Human, accession number HRA001329 (https://bigd.big.ac.cn/gsa-human/browse/HRA001329).

## Ethics Statement

The studies involving human participants were reviewed and approved by the Beijing Hospital Ethics Committee. Written informed consent to participate in this study was provided by the participants’ legal guardian/next of kin.

## Author Contributions

GQ, LW, and LY designed the study. GQ did the experiments. WZ, YJG, and YJF analyzed the scRNA-seq data. CZ, DY, MY, GY, and JJ checked the case history. LW and LY revise the manuscript. All authors contributed to the article and approved the submitted version.

## Conflict of Interest

The authors declare that the research was conducted in the absence of any commercial or financial relationships that could be construed as a potential conflict of interest.

## Publisher’s Note

All claims expressed in this article are solely those of the authors and do not necessarily represent those of their affiliated organizations, or those of the publisher, the editors and the reviewers. Any product that may be evaluated in this article, or claim that may be made by its manufacturer, is not guaranteed or endorsed by the publisher.

## Supplementary Material

Supplementary Figure 1 **(A)** tSNE of 8 samples. **(B)** Composition of 8 samples. The cell composition and cluster distribution were similar in 8 sample, which allowed for further analysis. **(C)** Feature plot of 16 cell markers.

Supplementary Figure 2 **(A)** Feature plot for CD8+ T/NK cell clusters. **(B**) Dotplot for CD4+ T cells.

Supplementary Figure 3 **(A)** GO analysis of DC. RAGE pathway can be found with higher expression in TA group in both CD14+ monocytes and DCs. **(B)** The comparation of ILs expression in each cell type between TA and Ctrl group.

Supplementary Figure 4 FACS results. **(A)** The flow charts of FACS. We used 9 following antibodies to confirm each cell type found in scRNA-seq: CD4+ T cell (CD45+ CD3+ CD4+), CD8+ T cell (CD45+ CD3+ CD8+), NKT cell (CD45+ CD3+ CD56+), B cell (CD45+ CD3- CD19+), NK cell (CD45+ CD3- CD56+), CD14+ monocyte (CD45+ CD3- CD14+ CD16-), CD16+ monocyte (CD45+ CD3- CD14+ CD16+), DC (CD45+ CD3- CD14- CD19- CD16- CD56- CD11C+). **(B)** The expression of CD163 on monocyte membrane. I, II, III, IV are TA group and V, VI, VII, VIII are Ctrl group. These figures show that the crests of curves in TA group are closer to the right side than Ctrl group, which means CD163 have a higher expression on TA patients’ monocyte surface. **(C)** The expression of CD163 on DC membrane. Similar to B, I, II, III, IV belong to TA group and V, VI, VII, VIII belong to Ctrl group. These figures show that CD163 express higher on TA patients’ DC surface.

Supplementary Figure 5 **(A)** qPCR results of 4 genes (TXNIP, AREG, THBS1, CD163). All genes were higher expressed in TA group. **(B)** ELISA results of 4 protein (TXNIP, AREG, THBS1, CD163). The serum levels of TXNIP and AREG were higher in TA group, however, THBS1 and CD163 were lower.

Supplementary Table 1 Clinical information for the peripheral blood mononuclear cells.

Supplementary Table 2 Cell markers for all cell clusters.

Supplementary Table 3 Cell markers for CD8+ T/NK cells.

Supplementary Table 4 Cell markers for CD4+ T cells.

Supplementary Table 5 DEGs for all cell types.
