# Supplementary figures and images for: Single-Cell RNA Sequencing Revealed CD14+ Monocytes Increased in Patients With Takayasu’s Arteritis Requiring Surgical Management
Source: Front Cell Dev Biol. 2021 Oct 4;9:761300. doi: 10.3389/fcell.2021.761300 (PMC8521054; doi:10.3389/fcell.2021.761300)

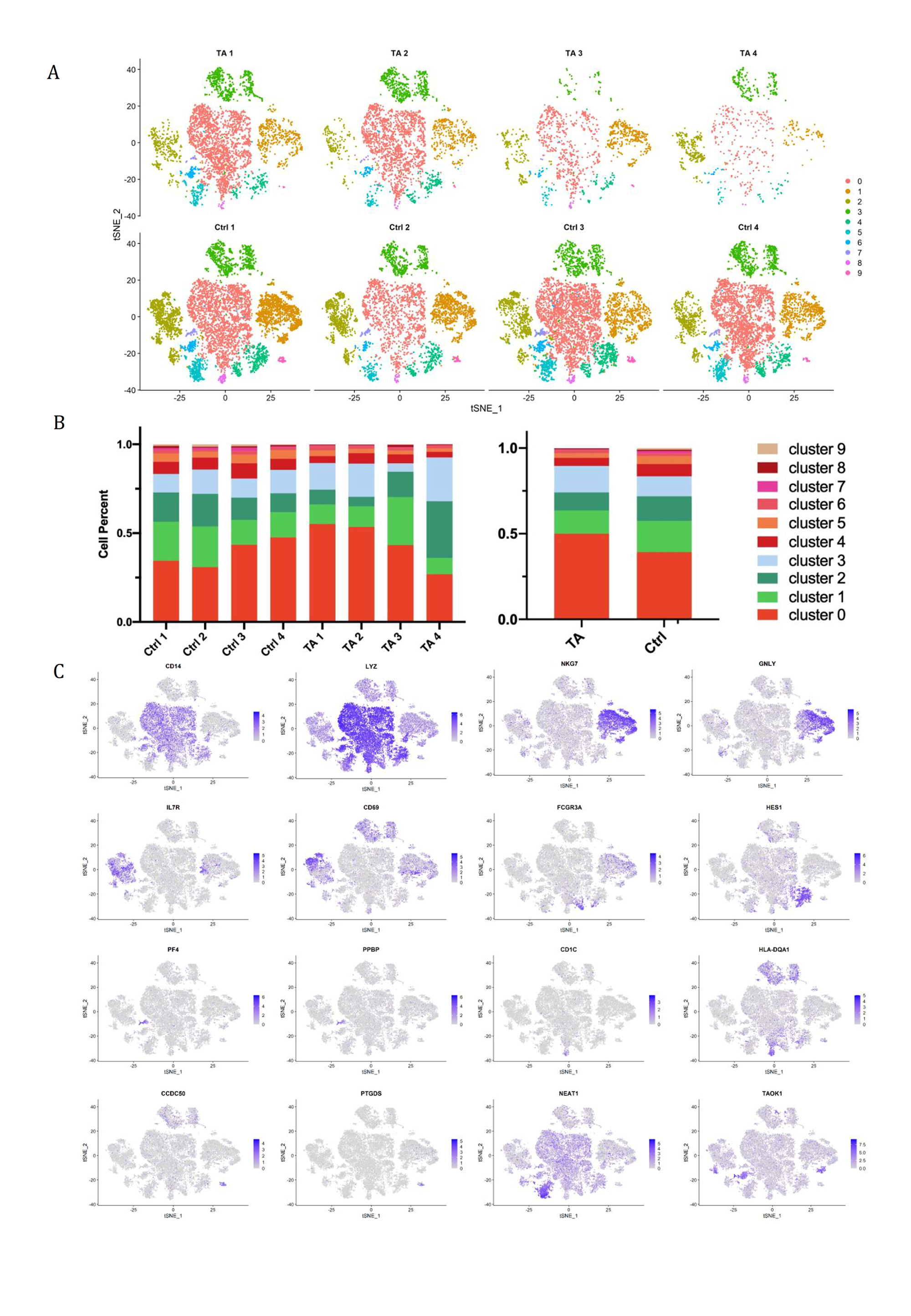

Supplement: Supplementary Figure 1 — (A) tSNE of 8 samples. (B) Composition of 8 samples. The cell composition and cluster distribution were similar in 8 sample, which allowed for further analysis. (C) Feature plot of 16 cell markers. [file Image_1.TIF]

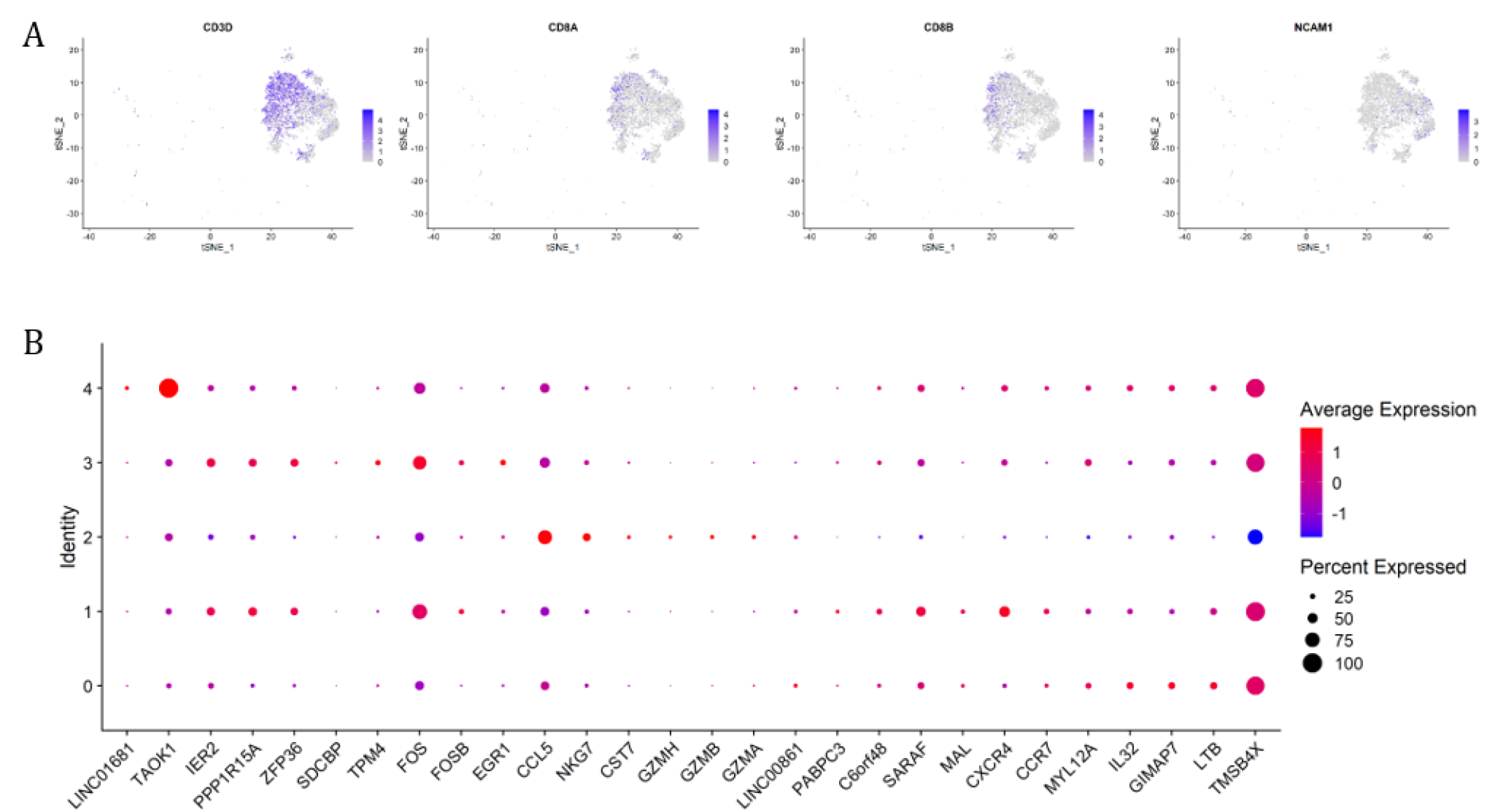

Supplement: Supplementary Figure 2 — (A) Feature plot for CD8+ T/NK cell clusters. (B) Dotplot for CD4+ T cells. [file Image_2.TIF]

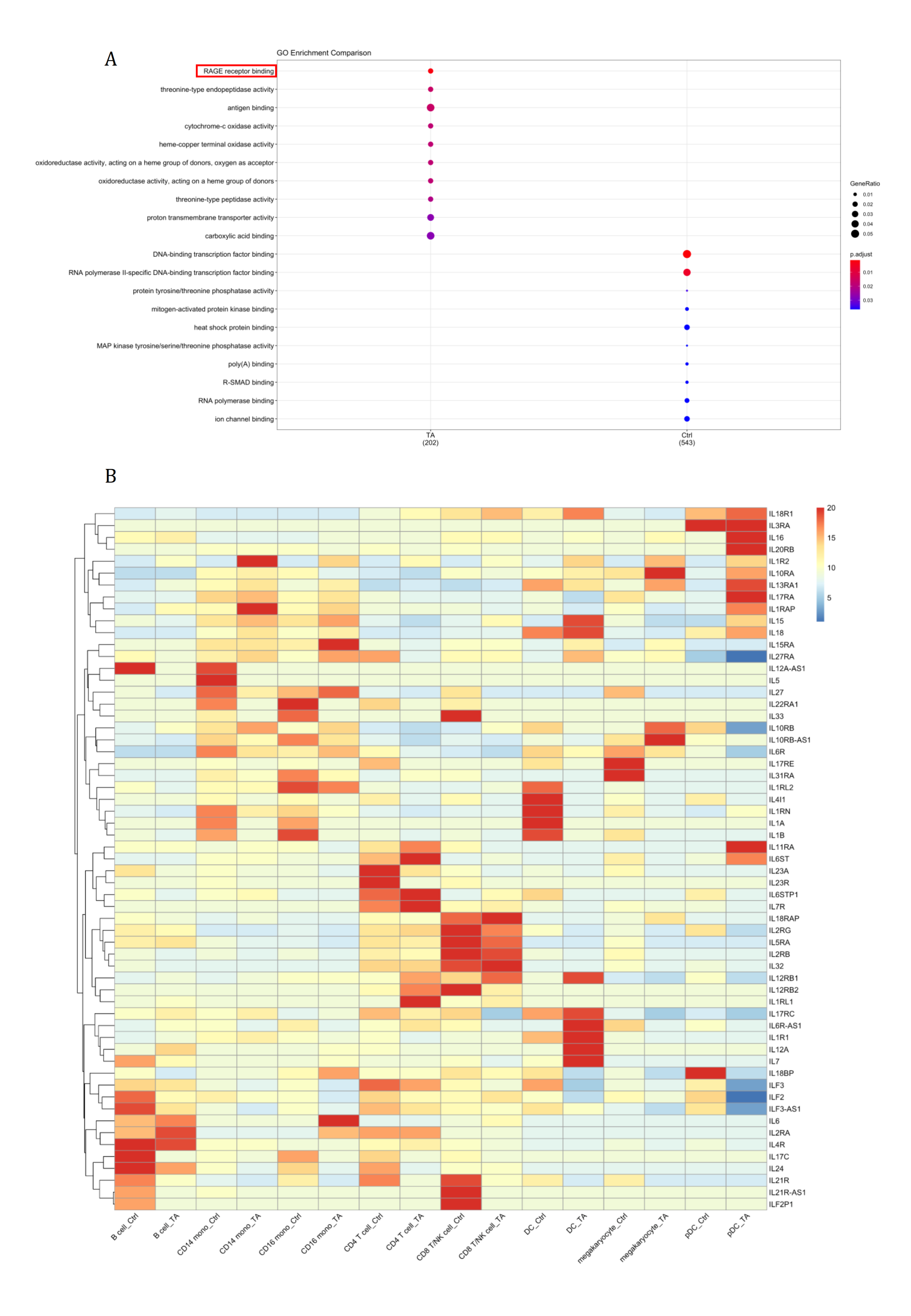

Supplement: Supplementary Figure 3 — (A) GO analysis of DC. RAGE pathway can be found with higher expression in TA group in both CD14+ monocytes and DCs. (B) The comparation of ILs expression in each cell type between TA and Ctrl group. [file Image_3.TIF]

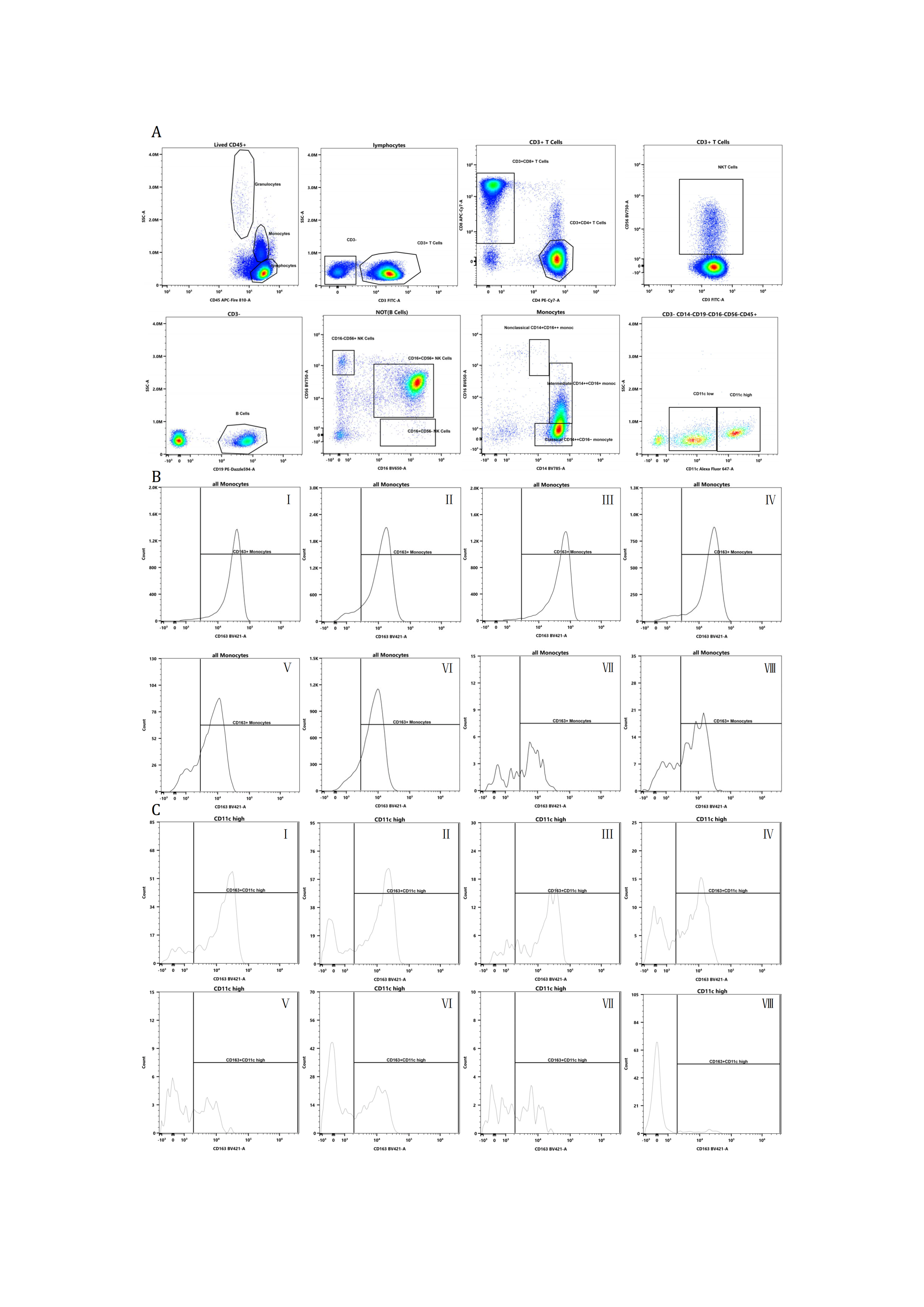

Supplement: Supplementary Figure 4 — FACS results. (A) The flow charts of FACS. We used 9 following antibodies to confirm each cell type found in scRNA-seq: CD4+ T cell (CD45+ CD3+ CD4+), CD8+ T cell (CD45+ CD3+ CD8+), NKT cell (CD45+ CD3+ CD56+), B cell (CD45+ CD3- CD19+), NK cell (CD45+ CD3- CD56+), CD14+ monocyte (CD45+ CD3- CD14+ CD16-), CD16+ monocyte (CD45+ CD3- CD14+ CD16+), DC (CD45+ CD3- CD14- CD19- CD16- CD56- CD11C+). (B) The expression of CD163 on monocyte membrane. I, II, III, IV are TA group and V, VI, VII, VIII are Ctrl group. These figures show that the crests of curves in TA group are closer to the right side than Ctrl group, which means CD163 have a higher expression on TA patients’ monocyte surface. (C) The expression of CD163 on DC membrane. Similar to B, I, II, III, IV belong to TA group and V, VI, VII, VIII belong to Ctrl group. These figures show that CD163 express higher on TA patients’ DC surface. [file Image_4.TIF]

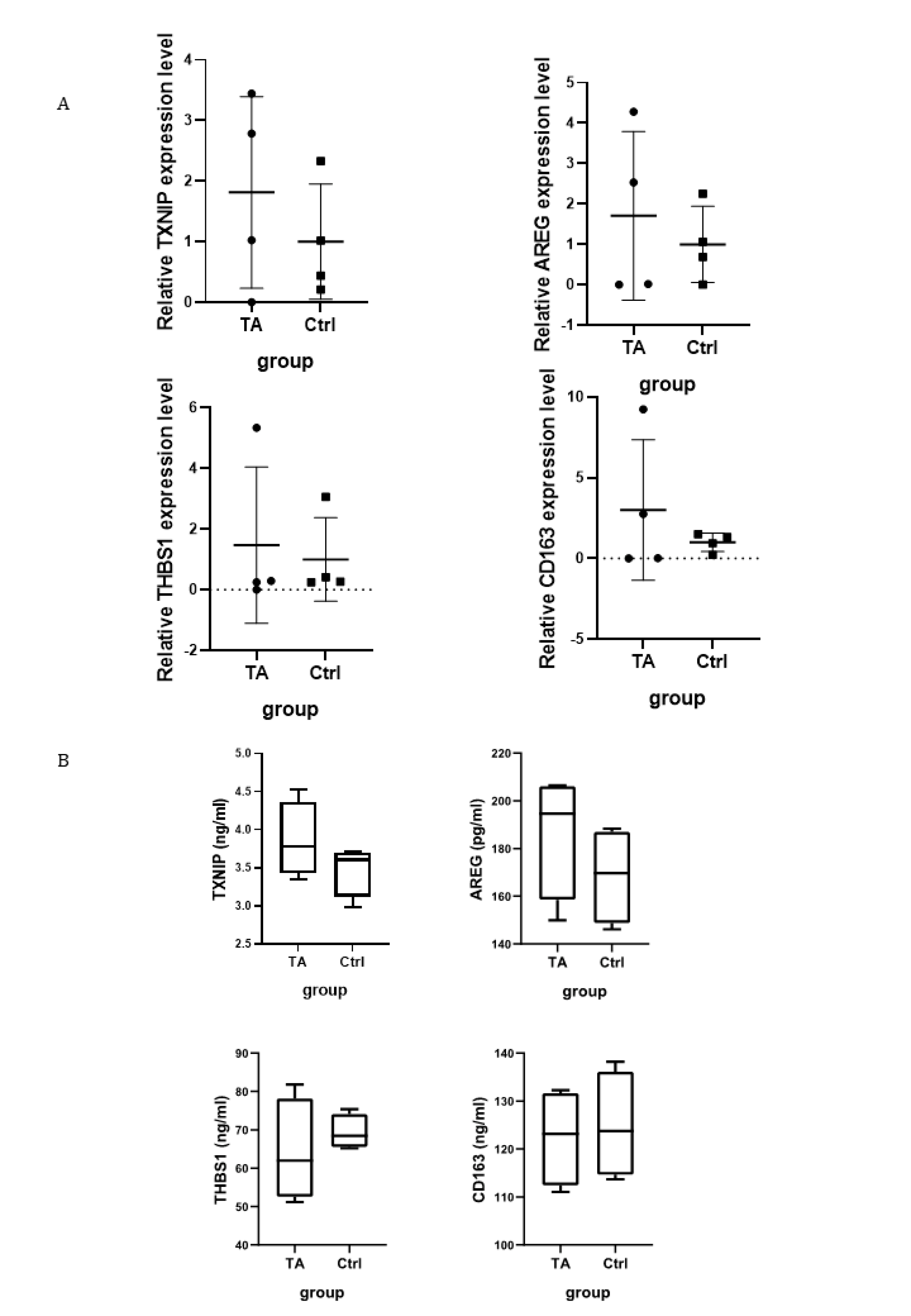

Supplement: Supplementary Figure 5 — (A) qPCR results of 4 genes (TXNIP, AREG, THBS1, CD163). All genes were higher expressed in TA group. (B) ELISA results of 4 protein (TXNIP, AREG, THBS1, CD163). The serum levels of TXNIP and AREG were higher in TA group, however, THBS1 and CD163 were lower. [file Image_5.TIF]
