# Supplementary material for: Single-Cell RNA Sequencing Revealed CD14+ Monocytes Increased in Patients With Takayasu’s Arteritis Requiring Surgical Management
Source: Front Cell Dev Biol. 2021 Oct 4;9:761300. doi: 10.3389/fcell.2021.761300 (PMC8521054; doi:10.3389/fcell.2021.761300)
Supplement: Supplementary Table 1 — Clinical information for the peripheral blood mononuclear cells. [file Table_1.DOCX]

| **Table S1. Clinical Information for the Peripheral Blood Mononuclear Cells** | | | | | | | | |
| --- | --- | --- | --- | --- | --- | --- | --- | --- |
| Variable | **TA1** | **TA2** | **TA3** | **TA4** | **Ctrl1** | **Ctrl2** | **Ctrl3** | **Ctrl4** |
| **Gender** | F | F | F | F | F | F | F | F |
| **Age, y** | 39 | 32 | 16 | 24 | 32 | 28 | 27 | 31 |
| **Diagnosis** | TA | TA | TA | TA | Donor | Donor | Donor | Donor |
| **Disease duration** | 7y | 4m | 3y | 4y | No | No | No | No |
| **Blood pressure (mmHg)** | 162/68 | 124/71 | 238/120 | 107/54 | No | No | No | No |
| **Dizzying** | Yes | Yes | Yes | No | No | No | No | No |
| **Blurred vision** | No | No | No | Yes | No | No | No | No |
| **Other cardiovascular disease** | No | CI | No | No | No | No | No | No |
| **Temperature** | 36.1 | 36.5 | 36.4 | 36.3 | No | No | No | No |
| **ESR (mm/hr)** | 5 | 5 | 7 | 7 | No | No | No | No |
| **CRP (mg/dL)** | <0.1 | 0.1 | 1.1 | 0.2 | No | No | No | No |
| **Vascular pain in the previous 3 months** | Yes | Yes | Yes | No | No | No | No | No |
| **Progressive process by CTA before operation** | Yes | Yes | Yes | No | No | No | No | No |
| **Drug use** | 6y | 4m | 3y | 4y | No | No | No | No |
| **PDN(mg/d)** | 0 | 0 | 17.5 | 0 | No | No | No | No |
| **MP (mg/d)** | 16 | 20 | 0 | 8 | No | No | No | No |
| **MTX (mg/d)** | 15 | 12.5 | 10 | 0 | No | No | No | No |
| **NIH criteria** | Active | Active | Active | Inactive | No | No | No | No |
| **Effected vessel** | CA+SA | CA+SA+MCA+ PCA | CT+SMA+RA+AA | CA+SA+VA | No | No | No | No |
| **Numano**  **Classification*** | Ⅰ | Ⅰ | Ⅲ | Ⅰ | No | No | No | No |
| Notes: PDN, Prednisone; MP, Methylprednisolone; MTX, Methotrexate; y, year; m, month; CI, Cerebral infarction; CA, Carotid artery; SA, Subclavian artery; MCA, Middle cerebral artery; PCA, Posterior cerebral artery; CT, coeliac trunk; SMA, Superior mesenteric artery; RA, Renal artery; AA, Abdominal aorta; VA, Vertebral artery | | | | | | | | |
