# Supplementary material for: Single-Cell RNA Sequencing Revealed CD14+ Monocytes Increased in Patients With Takayasu’s Arteritis Requiring Surgical Management
Source: Front Cell Dev Biol. 2021 Oct 4;9:761300. doi: 10.3389/fcell.2021.761300 (PMC8521054; doi:10.3389/fcell.2021.761300)
Supplement: Supplementary Table 2 — Cell markers for all cell clusters. [file Table_2.DOCX]

**Table S2 Cell markers for all cell clusters**

| **Gene** | **p_val** | **avg_log2FC** | **pct.1** | **pct.2** | **p_val_adj** | **cluster** |
| --- | --- | --- | --- | --- | --- | --- |
| S100A8 | 0 | 2.443686 | 0.984 | 0.605 | 0 | 0 |
| LYZ | 0 | 2.22018 | 0.996 | 0.659 | 0 | 0 |
| S100A9 | 0 | 2.194725 | 0.995 | 0.799 | 0 | 0 |
| CXCL8 | 0 | 2.008728 | 0.574 | 0.228 | 0 | 0 |
| CD14 | 0 | 1.830783 | 0.6 | 0.142 | 0 | 0 |
| CCL5 | 0 | 4.203671 | 0.968 | 0.475 | 0 | 1 |
| NKG7 | 0 | 3.94522 | 0.919 | 0.208 | 0 | 1 |
| GNLY | 0 | 3.800889 | 0.748 | 0.148 | 0 | 1 |
| GZMA | 0 | 3.104607 | 0.558 | 0.066 | 0 | 1 |
| GZMH | 0 | 2.918882 | 0.438 | 0.05 | 0 | 1 |
| IL7R | 0 | 2.967786 | 0.563 | 0.077 | 0 | 2 |
| CD69 | 0 | 2.107734 | 0.517 | 0.2 | 0 | 2 |
| MAL | 0 | 1.83654 | 0.163 | 0.011 | 0 | 2 |
| CCR7 | 0 | 1.814536 | 0.196 | 0.024 | 0 | 2 |
| MS4A1 | 0 | 4.584895 | 0.835 | 0.061 | 0 | 3 |
| IGHM | 0 | 4.255821 | 0.57 | 0.042 | 0 | 3 |
| CD79A | 0 | 3.006606 | 0.441 | 0.024 | 0 | 3 |
| CD79B | 0 | 2.596126 | 0.367 | 0.037 | 0 | 3 |
| HES1 | 0 | 4.245135 | 0.865 | 0.127 | 0 | 4 |
| HES4 | 0 | 1.684535 | 0.342 | 0.057 | 0 | 4 |
| CDKN1C | 0 | 1.435148 | 0.187 | 0.013 | 0 | 4 |
| FCGR3A | 1.91E-246 | 1.326547 | 0.302 | 0.076 | 4.26E-242 | 4 |
| NEAT1 | 0 | 3.446444 | 0.935 | 0.355 | 0 | 5 |
| XIST | 0 | 1.814155 | 0.483 | 0.118 | 0 | 5 |
| TAOK1 | 0 | 5.726592 | 1 | 0.357 | 0 | 6 |
| LINC01681 | 3.25E-119 | 4.938284 | 0.343 | 0.078 | 7.25E-115 | 6 |
| PF4 | 0 | 5.559272 | 0.971 | 0.044 | 0 | 7 |
| PPBP | 0 | 5.185928 | 0.806 | 0.05 | 0 | 7 |
| CD1C | 0 | 2.323557 | 0.508 | 0.018 | 0 | 8 |
| HLA-DQA1 | 1.21E-276 | 3.208042 | 0.963 | 0.174 | 2.70E-272 | 8 |
| HLA-DPB1 | 1.93E-177 | 2.966216 | 0.992 | 0.373 | 4.31E-173 | 8 |
| HLA-DPA1 | 5.21E-162 | 2.74877 | 0.988 | 0.4 | 1.16E-157 | 8 |
| PTGDS | 0 | 4.057225 | 0.408 | 0.012 | 0 | 9 |
| ITM2C | 0 | 3.870066 | 0.811 | 0.023 | 0 | 9 |
| TCF4 | 0 | 3.29367 | 0.786 | 0.047 | 0 | 9 |
| CCDC50 | 0 | 3.251211 | 0.782 | 0.061 | 0 | 9 |
