# Supplementary material for: Single-Cell RNA Sequencing Revealed CD14+ Monocytes Increased in Patients With Takayasu’s Arteritis Requiring Surgical Management
Source: Front Cell Dev Biol. 2021 Oct 4;9:761300. doi: 10.3389/fcell.2021.761300 (PMC8521054; doi:10.3389/fcell.2021.761300)
Supplement: Supplementary Table 3 — Cell markers for CD8+ T/NK cells. [file Table_3.DOCX]

**Table S3 Cell markers for CD8^+^ T/NK cells**

| **Gene** | **p_val** | **avg_log2FC** | **pct.1** | **pct.2** | **p_val_adj** | **cluster** |
| --- | --- | --- | --- | --- | --- | --- |
| CD3G | 1.51E-122 | 1.296022 | 0.586 | 0.257 | 3.36E-118 | 0 |
| CD3D | 8.90E-102 | 1.198459 | 0.539 | 0.245 | 1.98E-97 | 0 |
| TRAC | 3.01E-87 | 1.536965 | 0.316 | 0.089 | 6.70E-83 | 0 |
| TRBC2 | 4.69E-42 | 0.844286 | 0.551 | 0.382 | 1.04E-37 | 0 |
| CD8B | 1.16E-40 | 1.246009 | 0.136 | 0.029 | 2.58E-36 | 0 |
| TRGC2 | 1.68E-39 | 1.060571 | 0.245 | 0.104 | 3.74E-35 | 0 |
| IL7R | 5.58E-35 | 1.200706 | 0.243 | 0.111 | 1.24E-30 | 0 |
| CD8A | 4.81E-24 | 0.935511 | 0.132 | 0.049 | 1.07E-19 | 0 |
| GNLY | 8.77E-215 | 1.238131 | 0.916 | 0.64 | 1.95E-210 | 1 |
| CLIC3 | 4.18E-55 | 1.233646 | 0.269 | 0.103 | 9.32E-51 | 1 |
| KLRF1 | 2.32E-63 | 1.229422 | 0.309 | 0.122 | 5.17E-59 | 1 |
| PRF1 | 2.20E-64 | 1.149608 | 0.418 | 0.213 | 4.90E-60 | 1 |
| CD7 | 2.52E-58 | 1.061177 | 0.406 | 0.212 | 5.61E-54 | 1 |
| SPON2 | 4.00E-40 | 1.01285 | 0.26 | 0.119 | 8.92E-36 | 1 |
| CD160 | 8.89E-41 | 1.002439 | 0.168 | 0.053 | 1.98E-36 | 1 |
| CXXC5 | 5.31E-28 | 0.985286 | 0.169 | 0.073 | 1.18E-23 | 1 |
| TAOK1 | 1.77E-273 | 5.480877 | 1 | 0.301 | 3.95E-269 | 2 |
| LINC01681 | 8.86E-26 | 1.933827 | 0.239 | 0.075 | 1.97E-21 | 2 |
| SPATA22 | 3.00E-24 | 1.748778 | 0.107 | 0.018 | 6.69E-20 | 2 |
