# Supplementary material for: Single-Cell RNA Sequencing Revealed CD14+ Monocytes Increased in Patients With Takayasu’s Arteritis Requiring Surgical Management
Source: Front Cell Dev Biol. 2021 Oct 4;9:761300. doi: 10.3389/fcell.2021.761300 (PMC8521054; doi:10.3389/fcell.2021.761300)
Supplement: Supplementary Table 4 — Cell markers for CD4+ T cells. [file Table_4.DOCX]

**Table S4 Cell markers for CD4^+^ T cells**

| **Gene** | **p_val** | **avg_log2FC** | **pct.1** | **pct.2** | **p_val_adj** | **cluster** |
| --- | --- | --- | --- | --- | --- | --- |
| GIMAP7 | 1.93E-16 | 0.88629 | 0.353 | 0.261 | 4.30E-12 | 0 |
| LTB | 1.07E-15 | 0.883913 | 0.366 | 0.278 | 2.39E-11 | 0 |
| IL32 | 1.14E-18 | 0.876729 | 0.348 | 0.243 | 2.55E-14 | 0 |
| CCR7 | 5.86E-12 | 0.333024 | 0.273 | 0.167 | 1.31E-07 | 1 |
| CXCR4 | 1.97E-76 | 1.177221 | 0.536 | 0.228 | 4.40E-72 | 1 |
| MAL | 4.49E-08 | 0.279357 | 0.22 | 0.142 | 0.000999 | 1 |
| SARAF | 7.95E-17 | 0.415256 | 0.495 | 0.328 | 1.77E-12 | 1 |
| C6orf48 | 2.01E-14 | 0.340422 | 0.3 | 0.176 | 4.48E-10 | 1 |
| GZMB | 1.26E-39 | 1.575397 | 0.211 | 0.052 | 2.81E-35 | 2 |
| GZMH | 2.81E-28 | 1.306536 | 0.17 | 0.046 | 6.25E-24 | 2 |
| GZMA | 7.32E-17 | 0.846477 | 0.201 | 0.083 | 1.63E-12 | 2 |
| CCL5 | 1.89E-44 | 1.129382 | 0.727 | 0.457 | 4.21E-40 | 2 |
| NKG7 | 3.12E-27 | 1.199607 | 0.408 | 0.207 | 6.96E-23 | 2 |
| EGR1 | 3.11E-09 | 0.729762 | 0.272 | 0.145 | 6.94E-05 | 3 |
| FOS.3 | 9.60E-17 | 0.968195 | 0.681 | 0.551 | 2.14E-12 | 3 |
| FOSB.2 | 4.65E-06 | 0.397819 | 0.251 | 0.15 | 0.1037 | 3 |
| ZFP36.3 | 9.65E-12 | 0.760808 | 0.394 | 0.231 | 2.15E-07 | 3 |
| IER2.2 | 0.000135 | 0.326192 | 0.442 | 0.326 | 1 | 3 |
| TAOK1.2 | 5.91E-216 | 4.916322 | 1 | 0.299 | 1.32E-211 | 4 |
| LINC01681 | 5.46E-14 | 2.612394 | 0.198 | 0.072 | 1.22E-09 | 4 |
