# Supplementary material for: Single-Cell RNA Sequencing Revealed CD14+ Monocytes Increased in Patients With Takayasu’s Arteritis Requiring Surgical Management
Source: Front Cell Dev Biol. 2021 Oct 4;9:761300. doi: 10.3389/fcell.2021.761300 (PMC8521054; doi:10.3389/fcell.2021.761300)
Supplement: Supplementary Table 5 — DEGs for all cell types. [file Table_5.DOCX]

**Table S5 DEGs for all cell types**

| **Gene** | **p_val** | **avg_log2FC** | **pct.1** | **pct.2** | **p_val_adj** |
| --- | --- | --- | --- | --- | --- |
| IL1R2 | 0 | 2.959541 | 0.314 | 0.005 | 0 |
| THBS1 | 0 | 2.099411 | 0.186 | 0.013 | 0 |
| CD163 | 0 | 1.871044 | 0.299 | 0.049 | 0 |
| FKBP5 | 0 | 1.73358 | 0.385 | 0.114 | 0 |
| AREG | 0 | 1.563747 | 0.239 | 0.088 | 0 |
| CLEC4E | 0 | 1.468851 | 0.378 | 0.194 | 0 |
| MT-TP | 0 | 1.467274 | 0.444 | 0.327 | 0 |
| FTLP3 | 0 | 1.448243 | 0.363 | 0.178 | 0 |
| CEBPD | 0 | 1.273822 | 0.445 | 0.3 | 0 |
| AL365357.1 | 2.01E-267 | 1.267518 | 0.204 | 0.105 | 4.48E-263 |
| ZBTB16 | 0 | 1.246263 | 0.166 | 0.029 | 0 |
| MTND1P23 | 1.29E-260 | 1.227852 | 0.151 | 0.068 | 2.88E-256 |
| SMAP2 | 0 | 1.150314 | 0.536 | 0.371 | 0 |
| TXNIP | 0 | 1.148789 | 0.877 | 0.771 | 0 |
| AC090498.1 | 6.61E-233 | 1.133964 | 0.217 | 0.106 | 1.47E-228 |
| MTATP6P1 | 0 | 1.133608 | 0.805 | 0.706 | 0 |
| MT-TE | 0 | 1.123531 | 0.325 | 0.259 | 0 |
| HMGB2 | 2.03E-307 | 1.083038 | 0.31 | 0.184 | 4.53E-303 |
| IFITM1 | 2.47E-161 | 1.031817 | 0.112 | 0.071 | 5.50E-157 |
| H3F3A | 0 | 1.023337 | 0.403 | 0.285 | 0 |
| AL355472.2 | 1.10E-215 | 1.014746 | 0.143 | 0.043 | 2.45E-211 |
| MIF | 7.52E-173 | 1.011558 | 0.144 | 0.057 | 1.68E-168 |
| CXCR4 | 1.66E-247 | 0.980041 | 0.36 | 0.224 | 3.69E-243 |
| IRS2 | 9.85E-207 | 0.979802 | 0.189 | 0.102 | 2.20E-202 |
| MT2A | 1.66E-150 | 0.958654 | 0.241 | 0.171 | 3.69E-146 |
| MTCO1P12 | 0 | 0.956376 | 0.43 | 0.307 | 0 |
| SAMSN1 | 1.42E-197 | 0.938285 | 0.18 | 0.078 | 3.16E-193 |
| ZFP36L2 | 0 | 0.936765 | 0.5 | 0.425 | 0 |
| IGLC2 | 2.22E-35 | 0.927482 | 0.131 | 0.082 | 4.95E-31 |
| UPP1 | 1.17E-192 | 0.882721 | 0.272 | 0.166 | 2.62E-188 |
| GPX1P1 | 6.57395134679037e-312 | 0.872902 | 0.124 | 0.018 | 1.46E-307 |
| TSC22D3 | 2.60E-275 | 0.869857 | 0.478 | 0.35 | 5.79E-271 |
| NUDT16 | 1.19E-191 | 0.850038 | 0.161 | 0.069 | 2.65E-187 |
| CALM2 | 0 | 0.834958 | 0.458 | 0.374 | 0 |
| S100A12 | 2.67E-206 | 0.831982 | 0.439 | 0.365 | 5.96E-202 |
| CPM | 5.04E-145 | 0.737016 | 0.107 | 0.036 | 1.12E-140 |
| SPTLC2 | 3.86E-140 | 0.731682 | 0.176 | 0.102 | 8.60E-136 |
| MEGF9 | 6.97E-176 | 0.729532 | 0.264 | 0.209 | 1.55E-171 |
| FLII | 1.09E-140 | 0.710036 | 0.208 | 0.151 | 2.42E-136 |
| AC115223.1 | 1.44E-102 | 0.706495 | 0.173 | 0.113 | 3.20E-98 |
| AL138785.1 | 1.18E-89 | 0.705097 | 0.105 | 0.052 | 2.64E-85 |
| KLF9 | 1.60E-93 | 0.704826 | 0.175 | 0.103 | 3.56E-89 |
| AC005912.1 | 2.85E-206 | 0.692123 | 0.53 | 0.509 | 6.35E-202 |
| ETS2 | 1.08E-125 | 0.688663 | 0.161 | 0.097 | 2.40E-121 |
| CSGALNACT2 | 1.28E-132 | 0.677209 | 0.234 | 0.16 | 2.84E-128 |
| ASPH | 7.87E-114 | 0.669027 | 0.107 | 0.043 | 1.75E-109 |
| FTH1P8 | 1.29E-161 | 0.668046 | 0.206 | 0.146 | 2.88E-157 |
| KRAS | 9.90E-85 | 0.654542 | 0.134 | 0.108 | 2.20E-80 |
| IFNGR1 | 8.66E-118 | 0.652857 | 0.172 | 0.106 | 1.93E-113 |
| IGKC | 7.09E-43 | 0.651461 | 0.218 | 0.151 | 1.58E-38 |
| MTCO3P12 | 2.89E-93 | 0.645193 | 0.146 | 0.084 | 6.44E-89 |
| FTH1P10 | 3.37E-200 | 0.640745 | 0.285 | 0.239 | 7.51E-196 |
| RETN | 1.08E-91 | 0.619781 | 0.118 | 0.056 | 2.41E-87 |
| AL034397.3 | 1.45E-94 | 0.616421 | 0.109 | 0.054 | 3.24E-90 |
| RHOB | 9.53E-114 | 0.609288 | 0.168 | 0.125 | 2.12E-109 |
| CDC42P6 | 1.28E-88 | 0.60104 | 0.137 | 0.082 | 2.84E-84 |
| SERPINB1 | 2.86E-165 | 0.597491 | 0.316 | 0.272 | 6.38E-161 |
| FTH1P20 | 7.90E-116 | 0.594278 | 0.122 | 0.068 | 1.76E-111 |
| VCAN | 3.23E-191 | 0.59135 | 0.551 | 0.497 | 7.19E-187 |
| IGHM | 1.28E-18 | 0.588996 | 0.117 | 0.107 | 2.85E-14 |
| NCF1 | 2.89E-93 | 0.575505 | 0.117 | 0.076 | 6.43E-89 |
| IGHA1 | 2.65E-40 | 0.570113 | 0.105 | 0.063 | 5.90E-36 |
| MT-TC | 7.12E-108 | 0.569228 | 0.101 | 0.075 | 1.59E-103 |
| AC136632.1 | 5.16E-77 | 0.569092 | 0.184 | 0.147 | 1.15E-72 |
| IRAK3 | 3.43E-91 | 0.562873 | 0.16 | 0.113 | 7.64E-87 |
| RAB31 | 1.13E-144 | 0.561587 | 0.28 | 0.243 | 2.52E-140 |
| RNASE6 | 1.90E-100 | 0.561474 | 0.169 | 0.13 | 4.24E-96 |
| SLA | 1.48E-75 | 0.553285 | 0.182 | 0.132 | 3.30E-71 |
| TMSB4XP8 | 1.74E-98 | 0.551255 | 0.137 | 0.112 | 3.87E-94 |
| MT-TW | 1.10E-113 | 0.546198 | 0.217 | 0.192 | 2.46E-109 |
| PER1 | 6.18E-73 | 0.539215 | 0.116 | 0.067 | 1.38E-68 |
| CYTIP | 4.98E-62 | 0.535723 | 0.148 | 0.111 | 1.11E-57 |
| PTMAP2 | 2.70E-218 | 0.533745 | 0.444 | 0.447 | 6.02E-214 |
| C1orf162 | 7.71E-180 | 0.532553 | 0.383 | 0.36 | 1.72E-175 |
| KCTD12 | 3.40E-109 | 0.530994 | 0.186 | 0.149 | 7.57E-105 |
| MPHOSPH6 | 6.85E-63 | 0.522643 | 0.103 | 0.069 | 1.53E-58 |
| METTL7A | 8.40E-71 | 0.521147 | 0.148 | 0.106 | 1.87E-66 |
| CSTA | 2.49E-161 | 0.517785 | 0.398 | 0.372 | 5.55E-157 |
| SLC25A37 | 8.44E-59 | 0.517156 | 0.121 | 0.092 | 1.88E-54 |
| MT-CO3 | 0 | 0.513798 | 0.982 | 0.987 | 0 |
| VSIR | 1.37E-182 | 0.510779 | 0.399 | 0.376 | 3.06E-178 |
| MT-TS2 | 5.54E-153 | 0.504187 | 0.182 | 0.189 | 1.23E-148 |
| PABPC1P4 | 1.19E-70 | 0.500184 | 0.115 | 0.083 | 2.65E-66 |
| MS4A6A | 1.49E-146 | 0.497761 | 0.398 | 0.36 | 3.32E-142 |
| AL590867.2 | 6.19E-142 | 0.497569 | 0.395 | 0.4 | 1.38E-137 |
| AC131235.1 | 6.56E-71 | 0.492541 | 0.189 | 0.174 | 1.46E-66 |
| HMGB1P5 | 2.83E-135 | 0.490261 | 0.328 | 0.323 | 6.31E-131 |
| S100A11 | 2.59E-184 | 0.490167 | 0.536 | 0.512 | 5.78E-180 |
| SLC11A1 | 5.84E-96 | 0.482736 | 0.24 | 0.212 | 1.30E-91 |
| HRH2 | 2.82E-62 | 0.482216 | 0.152 | 0.118 | 6.29E-58 |
| SNX10 | 7.91E-91 | 0.481359 | 0.232 | 0.2 | 1.76E-86 |
| GLUL | 5.40E-65 | 0.478156 | 0.136 | 0.099 | 1.20E-60 |
| EEF1B2P3 | 2.26E-39 | 0.472502 | 0.105 | 0.078 | 5.03E-35 |
| MT-ND3 | 0 | 0.470659 | 0.884 | 0.896 | 0 |
| FTH1P11 | 6.26E-79 | 0.46543 | 0.106 | 0.068 | 1.39E-74 |
| CTSB | 3.97E-137 | 0.463568 | 0.327 | 0.31 | 8.83E-133 |
| ATP5MPL | 7.01E-133 | 0.461761 | 0.388 | 0.373 | 1.56E-128 |
| MT-ND1 | 0 | 0.460741 | 0.897 | 0.907 | 0 |
| CNTRL | 2.35E-88 | 0.46074 | 0.307 | 0.282 | 5.24E-84 |
| RAC1P2 | 3.56E-79 | 0.459614 | 0.164 | 0.137 | 7.93E-75 |
| ZFAND5 | 1.36E-157 | 0.453574 | 0.339 | 0.35 | 3.03E-153 |
| ISG20 | 1.25E-45 | 0.452221 | 0.131 | 0.12 | 2.78E-41 |
| TSPAN14 | 1.82E-45 | 0.448327 | 0.131 | 0.097 | 4.05E-41 |
| MT-ND2 | 0 | 0.439573 | 0.937 | 0.952 | 0 |
| EVI2B | 4.17E-163 | 0.436993 | 0.482 | 0.477 | 9.29E-159 |
| MFSD1 | 8.91E-71 | 0.435008 | 0.166 | 0.139 | 1.99E-66 |
| PREX1 | 4.05E-58 | 0.434509 | 0.152 | 0.129 | 9.02E-54 |
| RBM3 | 5.16E-142 | 0.434101 | 0.377 | 0.367 | 1.15E-137 |
| GAPDH | 3.20E-293 | 0.433864 | 0.637 | 0.658 | 7.14E-289 |
| TLR4 | 8.96E-69 | 0.427784 | 0.12 | 0.097 | 2.00E-64 |
| PFDN5 | 2.13E-201 | 0.427155 | 0.618 | 0.623 | 4.74E-197 |
| PTEN | 3.64E-71 | 0.426512 | 0.176 | 0.152 | 8.12E-67 |
| FOXO3 | 6.50E-52 | 0.424788 | 0.103 | 0.077 | 1.45E-47 |
| PGD | 3.02E-74 | 0.424755 | 0.176 | 0.147 | 6.74E-70 |
| LRRC75A-AS1 | 8.92E-84 | 0.423946 | 0.279 | 0.27 | 1.99E-79 |
| VAMP5 | 2.84E-46 | 0.419906 | 0.136 | 0.108 | 6.32E-42 |
| ALOX5 | 1.08E-60 | 0.417851 | 0.122 | 0.096 | 2.41E-56 |
| CELF2 | 2.67E-59 | 0.414482 | 0.217 | 0.204 | 5.94E-55 |
| GCA | 4.55E-51 | 0.412985 | 0.155 | 0.125 | 1.01E-46 |
| SLC31A2 | 5.38E-57 | 0.409334 | 0.128 | 0.101 | 1.20E-52 |
| MT-TT | 5.69E-75 | 0.407266 | 0.133 | 0.129 | 1.27E-70 |
| H3F3AP4 | 5.19E-139 | 0.406443 | 0.334 | 0.335 | 1.16E-134 |
| PLP2 | 7.92E-97 | 0.40504 | 0.266 | 0.258 | 1.77E-92 |
| GRINA | 7.77E-69 | 0.39952 | 0.191 | 0.168 | 1.73E-64 |
| SULT1A1 | 2.18E-48 | 0.3994 | 0.127 | 0.083 | 4.86E-44 |
| CCNY | 4.79E-101 | 0.395962 | 0.21 | 0.212 | 1.07E-96 |
| S100A8 | 3.06E-127 | 0.394065 | 0.767 | 0.766 | 6.81E-123 |
| EEF1A1P5 | 4.24E-43 | 0.391703 | 0.116 | 0.097 | 9.44E-39 |
| ARL4A | 8.79E-64 | 0.391333 | 0.297 | 0.272 | 1.96E-59 |
| AC004453.1 | 2.68E-79 | 0.391175 | 0.211 | 0.22 | 5.97E-75 |
| MT-ND4 | 1.20E-297 | 0.387898 | 0.972 | 0.981 | 2.67E-293 |
| MT-ATP8 | 2.60E-202 | 0.386916 | 0.857 | 0.89 | 5.80E-198 |
| ZCCHC6 | 4.17E-81 | 0.384722 | 0.261 | 0.253 | 9.30E-77 |
| AC007969.1 | 3.63E-144 | 0.384616 | 0.364 | 0.393 | 8.09E-140 |
| HLA-F | 3.32E-82 | 0.38423 | 0.241 | 0.244 | 7.39E-78 |
| PLBD1 | 3.06E-78 | 0.381809 | 0.21 | 0.199 | 6.81E-74 |
| CSF3R | 7.18E-74 | 0.381775 | 0.293 | 0.282 | 1.60E-69 |
| UBA52 | 2.39E-224 | 0.381168 | 0.725 | 0.747 | 5.32E-220 |
| MT-ND5 | 1.05E-261 | 0.380256 | 0.911 | 0.929 | 2.33E-257 |
| AL049873.1 | 1.18E-86 | 0.375155 | 0.275 | 0.283 | 2.64E-82 |
| HEBP2 | 2.56E-73 | 0.371846 | 0.27 | 0.262 | 5.70E-69 |
| AP001324.1 | 6.50E-110 | 0.371768 | 0.316 | 0.347 | 1.45E-105 |
| SDCBP | 1.95E-61 | 0.370053 | 0.18 | 0.165 | 4.33E-57 |
| LTA4H | 6.66E-55 | 0.368516 | 0.165 | 0.148 | 1.48E-50 |
| MS4A1 | 7.22E-12 | 0.366242 | 0.169 | 0.157 | 1.61E-07 |
| RNF13 | 2.28E-58 | 0.364328 | 0.156 | 0.141 | 5.08E-54 |
| GBP2 | 1.06E-44 | 0.364144 | 0.157 | 0.14 | 2.36E-40 |
| S100A9 | 2.37E-168 | 0.363529 | 0.873 | 0.886 | 5.29E-164 |
| LILRB3 | 7.48E-65 | 0.358744 | 0.189 | 0.182 | 1.67E-60 |
| MT-ATP6 | 2.29E-290 | 0.358358 | 0.97 | 0.984 | 5.11E-286 |
| ALDH2 | 9.25E-57 | 0.350227 | 0.154 | 0.141 | 2.06E-52 |
| FGR | 1.11E-82 | 0.346894 | 0.241 | 0.236 | 2.48E-78 |
| SH3BGRL3 | 8.56E-232 | 0.346553 | 0.82 | 0.836 | 1.91E-227 |
| CD302 | 3.94E-83 | 0.346195 | 0.225 | 0.22 | 8.78E-79 |
| AC099560.2 | 7.24E-154 | 0.34545 | 0.602 | 0.672 | 1.61E-149 |
| DDIT4 | 1.63E-31 | 0.345122 | 0.111 | 0.098 | 3.64E-27 |
| P2RY13 | 9.06E-49 | 0.34382 | 0.134 | 0.118 | 2.02E-44 |
| CBX3 | 9.30E-136 | 0.341451 | 0.492 | 0.517 | 2.07E-131 |
| USP15 | 3.25E-105 | 0.337869 | 0.365 | 0.379 | 7.23E-101 |
| HADHA | 7.09E-78 | 0.337398 | 0.248 | 0.249 | 1.58E-73 |
| MT-TI | 2.74E-67 | 0.334845 | 0.125 | 0.122 | 6.11E-63 |
| LGALS1 | 5.53E-130 | 0.334466 | 0.563 | 0.564 | 1.23E-125 |
| AC087343.1 | 2.22E-55 | 0.333764 | 0.146 | 0.152 | 4.96E-51 |
| MT-ND4L | 1.27E-164 | 0.32706 | 0.748 | 0.784 | 2.82E-160 |
| WASF2 | 2.59E-70 | 0.32692 | 0.281 | 0.283 | 5.78E-66 |
| MT-CYB | 7.04E-219 | 0.326402 | 0.955 | 0.969 | 1.57E-214 |
| EEF1B2 | 9.69E-115 | 0.326382 | 0.498 | 0.541 | 2.16E-110 |
| ANXA2 | 5.16E-94 | 0.32589 | 0.31 | 0.317 | 1.15E-89 |
| NBPF26 | 2.28E-43 | 0.3231 | 0.13 | 0.12 | 5.08E-39 |
| SLC25A6 | 7.99E-163 | 0.32227 | 0.55 | 0.578 | 1.78E-158 |
| C6orf48 | 7.55E-62 | 0.321614 | 0.188 | 0.199 | 1.68E-57 |
| HSD17B11 | 2.51E-74 | 0.320662 | 0.221 | 0.224 | 5.60E-70 |
| TSPO | 4.50E-124 | 0.319545 | 0.469 | 0.478 | 1.00E-119 |
| ANAPC16 | 1.17E-57 | 0.317171 | 0.27 | 0.273 | 2.60E-53 |
| ASAH1 | 3.77E-93 | 0.316736 | 0.298 | 0.307 | 8.41E-89 |
| CST3 | 1.75E-131 | 0.31656 | 0.711 | 0.702 | 3.90E-127 |
| RIPOR2 | 4.51E-82 | 0.314556 | 0.357 | 0.368 | 1.00E-77 |
| IL13RA1 | 4.79E-37 | 0.314011 | 0.107 | 0.085 | 1.07E-32 |
| S100A6 | 2.35E-198 | 0.313438 | 0.696 | 0.729 | 5.25E-194 |
| S100A10 | 4.36E-133 | 0.312592 | 0.56 | 0.572 | 9.71E-129 |
| RNF149 | 6.33E-38 | 0.311643 | 0.151 | 0.138 | 1.41E-33 |
| YBX1P1 | 1.25E-35 | 0.309356 | 0.112 | 0.1 | 2.78E-31 |
| KIAA1551 | 1.07E-85 | 0.308966 | 0.193 | 0.222 | 2.39E-81 |
| SORL1 | 6.28E-37 | 0.30885 | 0.117 | 0.111 | 1.40E-32 |
| PARP4 | 5.29E-42 | 0.308425 | 0.152 | 0.146 | 1.18E-37 |
| LRRK2 | 9.84E-58 | 0.308312 | 0.165 | 0.158 | 2.19E-53 |
| DICER1 | 7.95E-34 | 0.308034 | 0.136 | 0.125 | 1.77E-29 |
| TALDO1 | 3.55E-88 | 0.303296 | 0.305 | 0.312 | 7.91E-84 |
| ZNF281 | 1.52E-44 | 0.300898 | 0.105 | 0.101 | 3.38E-40 |
| Z74021.1 | 3.13E-44 | 0.300574 | 0.159 | 0.165 | 6.96E-40 |
| ARPC5 | 6.23E-86 | 0.298611 | 0.348 | 0.358 | 1.39E-81 |
| RNF130 | 3.49E-66 | 0.298446 | 0.203 | 0.199 | 7.77E-62 |
| BX679664.3 | 1.76E-45 | 0.297561 | 0.113 | 0.116 | 3.91E-41 |
| CD53 | 9.12E-104 | 0.297363 | 0.304 | 0.331 | 2.03E-99 |
| NDUFB1 | 2.29E-64 | 0.297004 | 0.265 | 0.272 | 5.10E-60 |
| GPX1 | 3.81E-120 | 0.296661 | 0.55 | 0.559 | 8.48E-116 |
| WDFY4 | 2.70E-37 | 0.294824 | 0.098 | 0.102 | 6.01E-33 |
| ITM2B | 6.74E-154 | 0.293728 | 0.522 | 0.559 | 1.50E-149 |
| RBP7 | 6.88E-49 | 0.293429 | 0.148 | 0.144 | 1.53E-44 |
| VNN2 | 1.18E-59 | 0.292997 | 0.108 | 0.113 | 2.63E-55 |
| CAPNS1 | 6.90E-65 | 0.292147 | 0.233 | 0.233 | 1.54E-60 |
| RBX1 | 1.34E-29 | 0.292004 | 0.148 | 0.14 | 2.98E-25 |
| STK17B | 5.77E-108 | 0.291445 | 0.376 | 0.405 | 1.28E-103 |
| GLRX | 5.71E-60 | 0.290386 | 0.251 | 0.256 | 1.27E-55 |
| SMIM25 | 1.37E-30 | 0.289463 | 0.145 | 0.13 | 3.05E-26 |
| ROCK1 | 7.53E-74 | 0.28946 | 0.286 | 0.299 | 1.68E-69 |
| CCND3 | 1.38E-40 | 0.289001 | 0.173 | 0.175 | 3.08E-36 |
| AC116533.1 | 3.30E-107 | 0.288251 | 0.32 | 0.358 | 7.35E-103 |
| YPEL3 | 1.47E-45 | 0.286602 | 0.147 | 0.15 | 3.27E-41 |
| CTSH | 2.93E-55 | 0.285817 | 0.195 | 0.199 | 6.54E-51 |
| TBXAS1 | 2.66E-39 | 0.280344 | 0.117 | 0.106 | 5.93E-35 |
| COX4I1 | 1.12E-130 | 0.276961 | 0.468 | 0.505 | 2.50E-126 |
| BTF3 | 2.47E-123 | 0.276154 | 0.445 | 0.487 | 5.50E-119 |
| EAPP | 1.10E-29 | 0.275837 | 0.123 | 0.121 | 2.45E-25 |
| FCGRT | 2.38E-70 | 0.274825 | 0.221 | 0.226 | 5.30E-66 |
| DYNLT1 | 8.83E-32 | 0.274737 | 0.1 | 0.094 | 1.97E-27 |
| HLA-B | 1.37E-189 | 0.274339 | 0.845 | 0.879 | 3.06E-185 |
| DECR1 | 8.55E-47 | 0.273972 | 0.162 | 0.165 | 1.91E-42 |
| AGTRAP | 5.58E-55 | 0.273012 | 0.215 | 0.217 | 1.24E-50 |
| AC113935.1 | 1.92E-64 | 0.272923 | 0.131 | 0.146 | 4.28E-60 |
| NDUFC1 | 1.78E-31 | 0.272631 | 0.126 | 0.127 | 3.97E-27 |
| SELENOH | 1.07E-52 | 0.271767 | 0.159 | 0.173 | 2.39E-48 |
| RASSF2 | 1.78E-45 | 0.269974 | 0.115 | 0.113 | 3.96E-41 |
| IFITM3 | 3.32E-49 | 0.269496 | 0.493 | 0.48 | 7.40E-45 |
| CDA | 4.54E-34 | 0.268532 | 0.127 | 0.116 | 1.01E-29 |
| SEC62 | 5.10E-61 | 0.265939 | 0.266 | 0.288 | 1.14E-56 |
| NCF2 | 6.68E-80 | 0.265727 | 0.361 | 0.368 | 1.49E-75 |
| COX7C | 4.73E-143 | 0.265339 | 0.526 | 0.579 | 1.05E-138 |
| NACA | 7.32E-154 | 0.2652 | 0.539 | 0.588 | 1.63E-149 |
| CD37 | 3.21E-82 | 0.265079 | 0.389 | 0.423 | 7.15E-78 |
| BFAR | 5.00E-20 | 0.264103 | 0.12 | 0.112 | 1.11E-15 |
| EIF6 | 5.28E-28 | 0.26397 | 0.127 | 0.121 | 1.18E-23 |
| AHNAK | 2.64E-78 | 0.263954 | 0.237 | 0.256 | 5.88E-74 |
| CD14 | 4.64E-41 | 0.262334 | 0.346 | 0.333 | 1.03E-36 |
| SELL | 3.35E-74 | 0.262106 | 0.245 | 0.265 | 7.47E-70 |
| NCOA4 | 5.83E-58 | 0.260931 | 0.176 | 0.184 | 1.30E-53 |
| FIS1 | 2.78E-32 | 0.260927 | 0.123 | 0.127 | 6.20E-28 |
| SCPEP1 | 6.65E-52 | 0.260921 | 0.148 | 0.153 | 1.48E-47 |
| TKT | 9.09E-91 | 0.260648 | 0.377 | 0.389 | 2.03E-86 |
| PTMAP5 | 2.68E-133 | 0.259873 | 0.359 | 0.417 | 5.96E-129 |
| IFNGR2 | 3.65E-58 | 0.258194 | 0.15 | 0.154 | 8.14E-54 |
| RAB7A | 1.77E-35 | 0.25657 | 0.128 | 0.126 | 3.94E-31 |
| MT-CO1 | 2.63E-196 | 0.25568 | 0.988 | 0.997 | 5.87E-192 |
| MRPS21 | 4.42E-26 | 0.255519 | 0.144 | 0.142 | 9.84E-22 |
| WAC-AS1 | 2.37E-26 | 0.252309 | 0.105 | 0.098 | 5.29E-22 |
| HCLS1 | 3.05E-48 | 0.251279 | 0.197 | 0.204 | 6.79E-44 |
| SSH2 | 2.87E-31 | 0.250804 | 0.108 | 0.105 | 6.38E-27 |
